# SOLA: dissecting dose-response patterns in multi-omics data using a semi-supervised workflow

**DOI:** 10.3389/fgene.2024.1508521

**Published:** 2024-12-02

**Authors:** Wanxin Lai, You Song, Knut Erik Tollefsen, Torgeir R. Hvidsten

**Affiliations:** ^1^ Bioinformatics and Applied Statistics (BIAS), Faculty of Chemistry, Biotechnology and Food Science, Norwegian University of Life Sciences (NMBU), Akershus, Norway; ^2^ Norwegian Institute for Water Research (NIVA), Oslo, Norway; ^3^ Norwegian University of Life Sciences (NMBU), Akershus, Norway; ^4^ Centre for Environmental Radioactivity (CERAD), Faculty of Environmental Sciences and Natural Resource Management (MINA), Norwegian University of Life Sciences (NMBU), Akershus, Norway

**Keywords:** dose-response patterns, non-monotonic response, radiation effects, Daphnia magna, multiomics, network analysis, semi-supervised approach, adverse outcome pathway (AOP)

## Abstract

An increasing number of ecotoxicological studies have used omics-data to understand the dose-response patterns of environmental stressors. However, very few have investigated complex non-monotonic dose-response patterns with multi-omics data. In the present study, we developed a novel semi-supervised network analysis workflow as an alternative to benchmark dose (BMD) modelling. We utilised a previously published multi-omics dataset generated from *Daphnia magna* after chronic gamma radiation exposure to obtain novel knowledge on the dose-dependent effects of radiation. Our approach combines 1) unsupervised co-expression network analysis to group genes with similar dose responses into modules; 2) supervised classification of these modules by relevant response patterns; 3) reconstruction of regulatory networks based on transcription factor binding motifs to reveal the mechanistic underpinning of the modules; 4) differential co-expression network analysis to compare the discovered modules across two datasets with different exposure periods; and 5) pathway enrichment analysis to integrate transcriptomics and metabolomics data. Our method unveiled both known and novel effects of gamma radiation, provide insight into shifts in responses from low to high dose rates, and can be used as an alternative approach for multi-omics dose-response analysis in future. The workflow SOLA (Semi-supervised Omics Landscape Analysis) is available at https://gitlab.com/wanxin.lai/SOLA.git.

## Introduction

High-throughput analyses of biological effects, such as omics-analysis (e.g., genomics, transcriptomics, proteomics, metabolomics), play a key role in providing in-depth mechanistic knowledge, classification of a stressor’s mode of action (MoA), and biomarker discovery in ecotoxicological research (Brockmeier et al., 2017). Recent advances in deriving point of departure (POD) values from omics data as references for setting exposure/toxicity thresholds of environmental stressors (Thomas et al., 2012; Reynolds et al., 2020; Xia et al., 2020; Song et al., 2020; Alcaraz et al., 2022) also represent a promising application of omics-data to support hazard assessments of environmental stressors. Such targeted approaches can facilitate the collection of empirical support for established adverse outcome pathways (AOPs) (Song et al., 2023; Gomes et al., 2018; Song et al., 2020; Xia et al., 2020) and the identification of new AOPs (Brockmeier et al., 2017). Nonetheless, these approaches fit data with predefined statistical models and have missed the discovery of novel dose-response relationships.

Most methods for analysing omics data in field of toxicology start from established adverse outcome pathways (AOPs) with a set of predefined genes or pathways (Song et al., 2020), and can therefore be characterised as targeted and supervised. An alternative is unsupervised methods, such as weighted gene co-expression network analysis (WGCNA) (Langfelder and Horvath, 2008), that has been used to identify important pathways without prior knowledge. There is a lack of method that can start out explorative (unsupervised), and at the same time allow incorporation of prior knowledge about the specific experiment (e.g., molecular endpoints) or affected pathways (e.g., AOPs) when appropriate. Moreover, methods typically do not include approaches for comparing models (e.g., co-expression networks) across test conditions (e.g., dose-response patterns or temporal responses) nor do they allow the integration of other omics data (multi-omics) (Langfelder and Horvath, 2008; Jeremias et al., 2018).

In the present study, we re-analysed a previously published multi-omics (transcriptomics and metabolomics) dataset on the chronic effects of gamma radiation on the freshwater crustacean *Daphnia magna* (Song et al., 2023). We took advantage of previous advancements in the field to demonstrate how a semi-supervised analyses of multi-omics data can generate new insights into dose- and time-dependent responses in *D. magna* exposed to radiation. The main aims of this study were to: 1) Establish a novel data analytical workflow for dealing with complex multi-omics data in *D. magna*; 2) Demonstrate the usefulness of the new analytical pipeline for understanding complex dose-response patterns; and 3) Identify new pathways to support the expansion of the AOP network for radiation.

## Methods and materials

### Experiment

We analysed a multi-omics dataset consisting of both transcriptomics and metabolomics measurements from *D. magna* exposed to gamma radiation. In brief, groups of 10 animals were exposed to seven different dose rates of gamma radiation (0 (control), 0.4, 1, 4, 10, 40, 100 mGy/h) for a period of either four or 8 days. The radiation exposure period of 8 days covers the transitional stage of daphnids from juvenile to adulthood (visible and unreleased embryo) while the 4 days exposure period only covers the temporal change in the juvenile stage. Omics profiling was performed on replicated, pooled, groups resulting in three datasets of 28 samples (seven dose rates x 4 replicates): transcriptomics after 4 days of exposure, and transcriptomics and metabolomics after 8 days of exposure.

## Data

The GEO accession ID for the transcriptome datasets is GSE207246, uploaded by the Norwegian Institute of Water Research (Oslo, Norway). The same team also provided the metabolome data.

### Data analysis pipeline

The data analysis workflow described in this study is available at https://gitlab.com/wanxin.lai/SOLA.git and DiCE is available at https://gitlab.com/hvidsten-lab/DiCE. All data figures and statistical analysis were generated in R studio using R version x64 4.4.1. A schematic of the workflow is presented in Supplementary Figure S1.

### Data pre-processing

Raw counts from transcriptomics were normalised using Variance Stabilizing Transformation (vst) from DESeq2 (Love et al., 2014).

### Module identification

Gene modules were identified using WGCNA (Langfelder and Horvath, 2008). RNA-seq data from 4- and 8-days of exposure were processed using the function blockwiseModules with networkType = “signed” and TOMType = “signed.” The 4-day transcriptomics data required a soft threshold (β) of 9 to achieve scale-free topology (*R* ^2^ = 0.98, mean connectivity = 94, Supplementary Figure S2). For the 8-day data, β = 25 was used (*R* ^2^ = 0.909, mean connectivity = 9.87).

## Differentially expressed genes (DEGs)

DESeq2 was used to identify DEGs (adjusted *p* < 0.05) by fitting a linear model to the data, contrasting low and high dose-rate responses (0 vs. 1 and 1 vs. 100 mGy/h).

### Significant modules

Significant modules were selected based on the enrichment of DEGs in each module, using the Fisher Exact test.

### Mapping and annotation

Blast2GO (v4.1) was used for mapping and sequence annotation between *D. magna* and *D. melanogaster* (Conesa and Götz, 2008). Transcripts were converted to Entrez IDs.

### Pathway enrichment analysis

Entrez IDs from each module were loaded into ReactomePA (v1.38) for pathway enrichment analysis, with pathways considered significant at *p* < 0.05 (Croft et al., 2010; Yu and He, 2016).

### GO enrichment analysis

GO enrichment analysis of significant modules was done using the R package clusterProfiler, with significance set at *p* < 0.05 (Yu and Petyuk, 2012).

Significant modules were also analysed using BiNGO in Cytoscape (Maere et al., 2005; Shannon et al., 2003), with enriched GO terms (BH-FDR <0.05) visualised using EnrichmentMap (Merico et al., 2010), and annotated with AutoAnnotate (Kucera et al., 2016).

### TF motif enrichment analysis

Expressed transcripts from *D. magna* were converted to gene sequences using the command line tool: NCBI datasets (Coordinators, 2015). The 2000bp upstream region of each coding sequences were extracted with the unix package bedops S6 (v2.4.40) (Neph et al., 2012). The selected sequences were piped to the AME (Analysis of Motif Enrichment) algorithm (Buske et al., 2010) in the MEME suite (Bailey et al., 2015), which uses several fly motif database for motif predictions. Genes that did not belong to the selected modules were used as background for the statistical test. The *D. melanogaster* protein sequence of the transcription factor binding the over-represented motifs were searched against NCBI to find the ortholog sequences in *D. magna.* . The search was done using the software package OrthoFinder v2.3.3 (Emms and Kelly, 2019), with DIAMOND (v0.9.24) and the setting ‘ultra-sensitivity’.

### Differential metabolites

PCA was used to assess metabolome data quality. The R package limma was used to identify differential metabolites (adjusted *p* < 0.05) (Ritchie et al., 2015).

### DiCE

Differential co-expression was quantified using the following formula (Voigt et al., 2017):

• Conserved: $\frac{\text{abs}\left(C_{\exp1}+C_{\exp2}\right)}{2}$

• Specific: $\left(\text{abs}\left(\text{abs}\left(C_{\exp1}\right)-\text{abs}\left(C_{\exp2}\right)\right)\right.$

• Differentiated: $\frac{\left(\text{abs}\left(C_{\exp1}\right)+\text{abs}\left(C_{\exp2}\right)-\text{abs}\left(C_{\exp1}+C_{\exp2}\right)\right)}{2}$

where C is the correlation matrix, and exp1 and exp2 refers to the 4-day and 8-day data, respectively. Correlation thresholds for including links in the differential co-expression network was set to 0.9, 0.7 and 0.5 for conserved, specific and differentiated co-expression, respectively.

### Integrated multi-omics pathway enrichment analysis

*D. magna* was selected as the model organism in Paintomics4, using NCBI accession IDs for transcriptomics data and KEGG compound names for metabolomics data (Liu et al., 2022). Input entries consisted of significant modules corresponding to low-, high-, and linear dose-rate responsive groups, combined with DiCE genes (conserved, specific, and differentiated correlation).

## Results

### Multi-omics data analysis workflow

**FIGURE 1:**
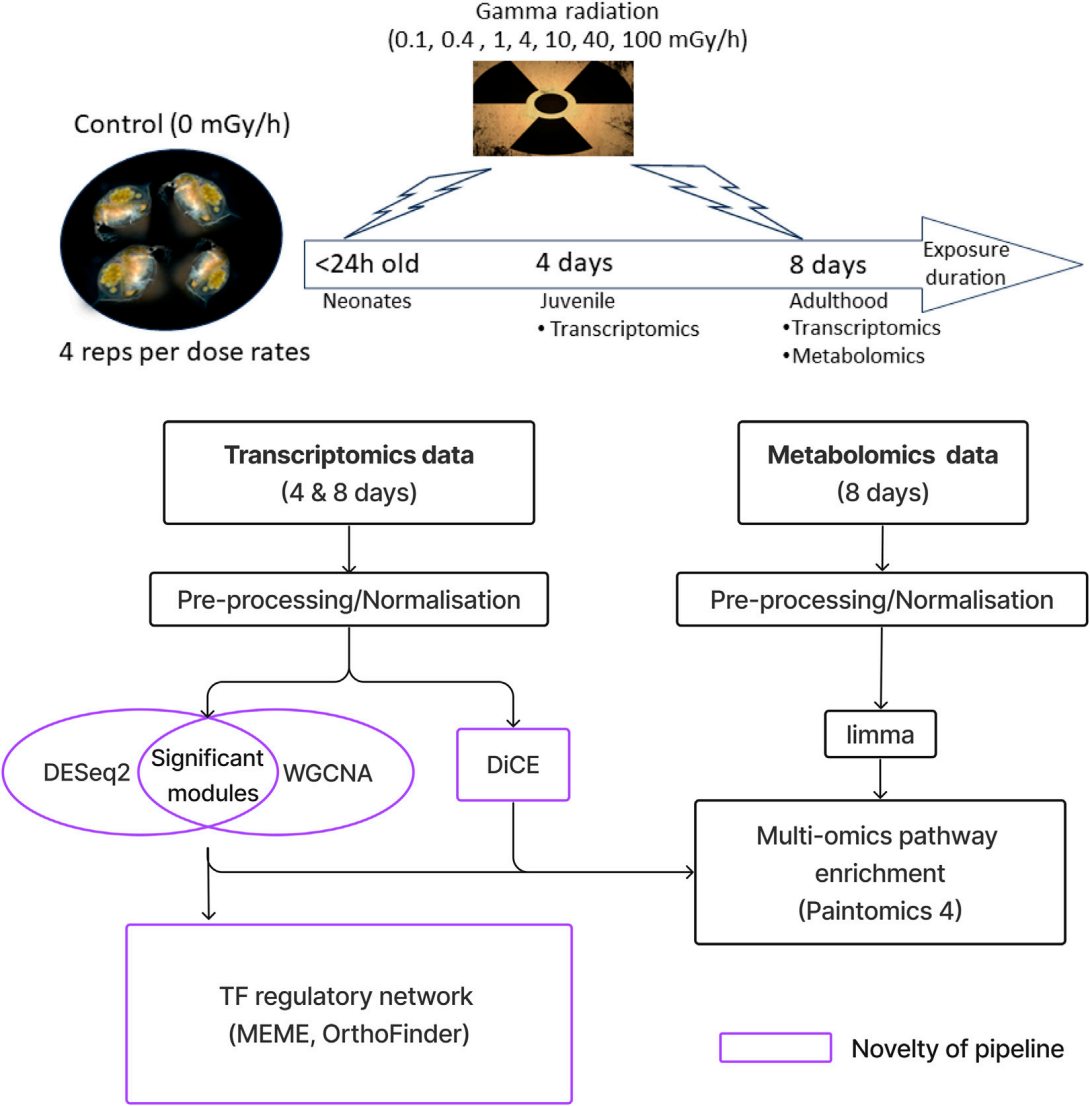
Workflow overview for the multi-omics data analysis. After normalization, co-expression network analysis (WGCNA) was used to organise genes into modules based on similarities in their expression profiles across dose-rates (dose response curves). These modules were then filtered based on statistically significant changes in gene expression (DESeq2) and functionally characterised (Gene Ontology and Reactome Pathway). To identify the regulatory mechanism driving the radiation responses, we reconstructed regulatory networks of the modules using transcription factor motifs from related species (using the MEME suite with the fly motifs in the FLY (*Drosophila melanogaster*) database; OrthoFinder was used to map transcription factors with motifs to D. magna). To compare the transcriptional response to gamma radiation between 4-day exposure and 8-day exposure, we used differential co-expression analysis (DiCE). The metabolomics data was used to identify metabolites with statistically significant changes in abundance across dose rates (limma). Differential metabolites and genes were then integrated to identify metabolic pathways affected by gamma radiation (Paintomics 4).

An overview of the experiment, the data and the data analysis workflow is presented in Figure 1 (details in Supplementary Figure S1). Co-expression network analysis (WGCNA) was used to group genes into modules based on similarities in their expression profiles across dose rates (dose response curves). These modules were then filtered based on statistically significant changes in gene expression (DEseq2) and functionally characterised (Gene Ontology (GO), Gene Ontology 2015; Reactome Pathway). To identify the regulatory mechanism driving the radiation responses, we reconstructed regulatory networks of the modules using transcription factor motifs from related species (MEME and OrthoFinder). To compare the transcriptional response to gamma radiation between 4-day exposure and 8-day exposure, we used differential co-expression analysis (DiCE). The metabolomics data was used to identify metabolites with statistically significant changes in abundance across dose rates (limma). Differential metabolites and genes were then integrated to identify metabolic pathways affected by gamma radiation (Paintomics4). The code is freely available at SOLA (Semi-supervised Landscapes Analysis). Taken together, this multi-omics workflow comprehensively describes and contrasts the molecular responses of *D. magna* to gamma radiation. Co-expression modules reveal diverse types of dose response relationships.

**FIGURE 2:**
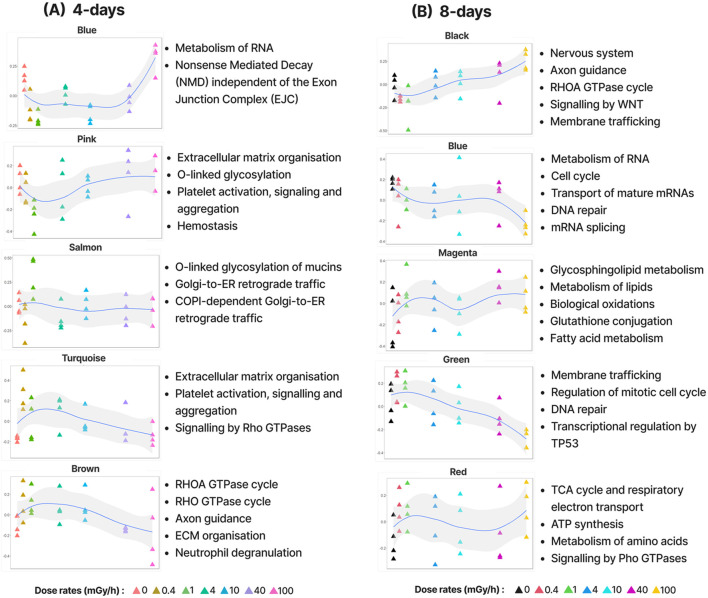
A few examples of significant modules from the **(A)** 4-day and **(B)** 8-day gamma radiation exposure data and their corresponding enriched Reactome pathways. Each module is represented by its eigengene (the first principal component of the module); *y*-axis showing the normalised eigengene value.

To describe the transcriptional changes in *D. magna* in response to different dose rates of gamma radiation, we used network analysis to group genes into modules based on similar expression profiles (co-expression). In total, out of 23,570 transcripts (21,549 genes), this analysis identified 38 modules containing 9,116 expressed genes after 4 days of exposure and 36 modules containing 8,309 expressed genes after 8 days of exposure (Supplementary Figure S2). To separate modules describing novel dose-rate dependent responses to radiation from those describing spurious variation in expression, differentially expressed genes (DEGs) were identified based on three types of DEG response patterns: genes with a monotonic increase or decrease in expression, genes with a low dose-rate response (0 vs. 1 mGy/h), and genes with a high dose-rate response (1 vs. 100 mGy/h) (Supplementary Table S1, Supplementary Figure S3). The gamma radiation dose rates of 1 and 100 mGy/h were used as the molecular endpoints (lowest dose rates that triggered molecular changes such as reactive oxygen species (ROS) formation) based on the preliminary discoveries by Song et al. (2020) and Gomes et al. (2018). They found that distinct mechanisms were triggered at varying dose rates, contributing to varying degrees of reduced fecundity in *D. magna*. Particularly, reproduction was delayed at the low dose rate (1 mGy/h), whereas at the high dose rate (100 mGy/h) the reproduction cycle was accelerated leading to smaller brood sizes and consequently reduced numbers of progenies. We classified modules as “significantly radiation responsive” if they were enriched in at least one type of DEG response pattern (Supplementary Figure S4). From this analysis, 9 and 13 modules from the 4 days and 8 days datasets were classified as significant, respectively (Figure 2; Supplementary Figures S4, S5). To functionally characterise the modules, we performed Reactome pathway enrichment (Reactome PA) and Gene Ontology (GO) analysis. 23% and 36% of the modules were enriched for at least one pathway in the 4- and 8-day exposure datasets, respectively (Supplementary Figure S6).

The modules found in the 4 and 8 days expose data reflected a wide range of expression patterns in response to increasing doses of gamma radiation (Figure 2). While the enriched pathways in the 4 days modules mostly were also enriched in the 8 days modules, the 8 days data revealed many pathways exclusive to long exposure time (GO analysis also supported this observation).

In the 4 days exposure data, the most distinct expression pattern was that of the **blue.4d** module, which contained genes that responded very specifically to high dose rates (100 mGy/h). This module was enriched for pathways involved in growth and development such as metabolism of RNA and translational regulation. In the 8 days exposure data, one of the most distinct patterns was the steady downregulation of genes in the **green.8d** module with increasing dose rates. This module was enriched for pathways such as DNA repair, “TP53 regulates metabolic genes” (key regulator of autophagy and apoptosis). These patterns indicate that various dose rates induce different cellular stress responses. At low to moderate dose rates, cells activate mechanisms aimed at repairing damage and maintaining cellular integrity. However, at higher doses, where damage exceeds the cell’s repair capacity, upregulation of genes involved in growth and development might ensure the production of essential proteins necessary for survival and recovery. Consistent with this interpretation, TP53, which is known to inhibit cell growth, is downregulated at high dose rates.

Taken together, these findings indicate that longer exposure to gamma radiation affected a greater range of molecular transport, cellular and developmental processes than shorter exposure (Supplementary Figure S6).

### Regulatory networks describe transcription factors mediating the transcriptional response to radiation

**FIGURE 3:**
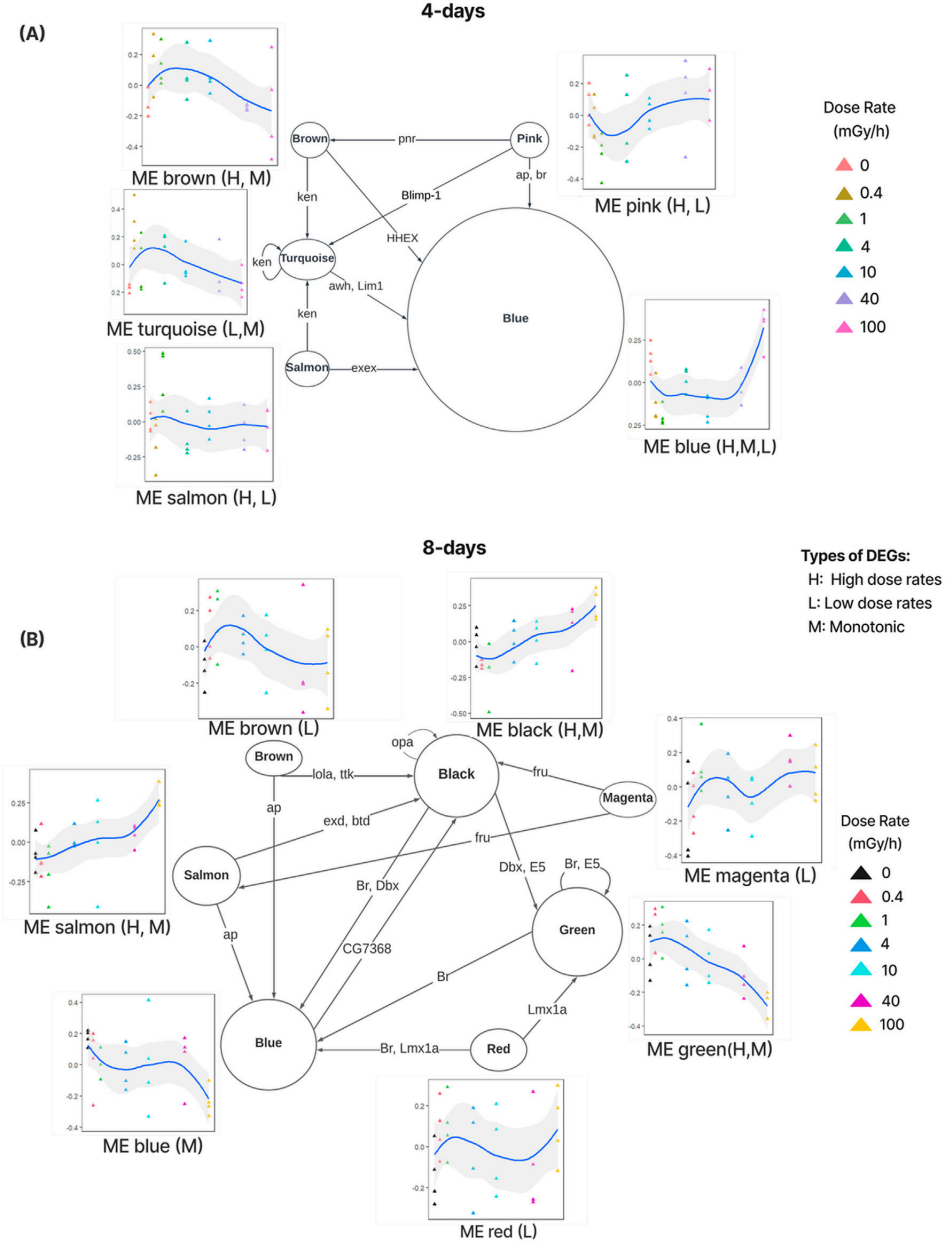
Regulatory networks showing transcription factors (TFs, labels on directed edges) located in significant module regulating genes in other significant module (where the TF’s motif is enriched) for 4 days **(A)** and 8 days **(B)** exposure data. The size of the nodes corresponds to the number of enriched motifs in that module. The expression profiles (eigengenes) of modules across dose rates. Three types of module patterns were identified based on overlap with the DEGs: monotonic (M) increase/decrease, low dose responsive (L) and high dose responsive (H).

To gain insight into the regulatory mechanisms underlying the transcriptional responses captured by the modules, regulatory networks were predicted by transferring transcription factor binding site (TFBS) information from related species. Briefly, if the DNA sequence pattern (motif) of the TFBSs of a specific TF was described in a related species, we searched for this pattern in the promoters of *D. magna* genes and predicted the matches to be bound by the *D. magna* ortholog of that TF (Supplementary Tables S2, S3). To describe regulatory mechanisms relevant to radiation response, the analysis was limited to significant modules and to TFs belonging to one of these modules (activated TFs). We predicted that the TF regulated a module if its motif was enriched in the promoters of genes belonging to that module (Figure 3).

For the 4 days exposure data, 21 activated TFs were identified of which 11 had enriched motifs in five significant modules (Figure 3A). For the 8 days exposure data, 39 activated TFs were identified of which 20 had enriched motifs in seven modules (Figure 3B).

Examining regulatory network **A**, the **pink.4d**, **brown.4d**, t**urquoise.4d** and **salmon.4d** modules are all predicted to contain TFs regulating genes in the **blue.4d** module. Noticeably, the genes in the **blue.4d** module are distinctly responsive to high dose rates (100 mGy/h), while the regulators typically are in modules activated by low dose rates. This suggests that regulators such as haematopoietically expressed homeobox (HHEX) in the **brown.4d** module inhibits the expression of genes in the **blue.4d** module. Interestingly, the GATA factor Pannier (Pnr) in the **pink.4d** module is also recognised as a haematopoietic regulator. *Pnr* plays a role in the formation of cardiac cells and the maturation of haemocytes in the lymph gland during the embryonic stage (Minakhina et al., 2011). As discussed earlier, the **blue.4d** module is enriched for pathways related to growth and development, which implies a shift towards survival and recovery at high dose rates. The regulatory network indicates that the activation of hematopoietic regulators may play a role in the underlying reprogramming of the transcriptome.

In the regulatory network **B**, a larger array of TF encoding genes were activated (Figure 3B). One interesting example is the activation of the TF encoding gene Lola at low dose rates (**brown.8d** module). The regulatory network predicts that Lola regulates genes in the **black.8d** module. These genes are enriched for pathways related to the nervous system and axon guidance and gradually increase in expression with rising dose rates (Figure 2). Supporting this prediction, ectopic expression of Lola has demonstrated a role in axon guidance and in preventing neurodegeneration (Dinges et al., 2017).

Taken together, our analysis of regulatory mechanism shows that longer exposure to radiation results in considerable reprogramming of the transcriptome mediated by TFs implicated in regeneration and damage repair.

### Differential network analysis reveals temporal response patterns

**FIGURE 4:**
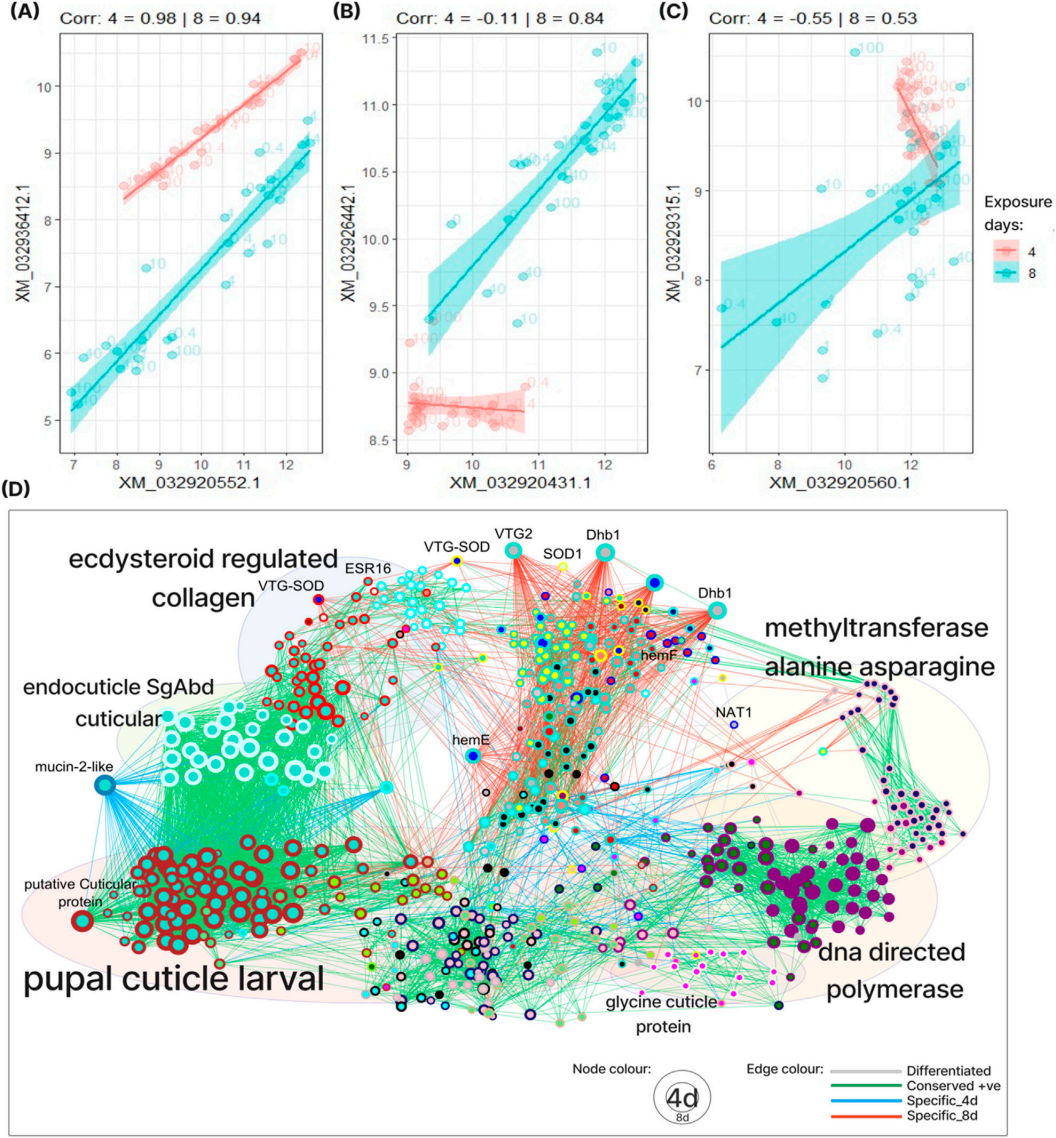
Differential co-expression analysis (DiCE) analysis. **(A–C)** Examples of the three types of differential co-expression described by the DiCE network: conserved **(A)**, specific **(B)** and differential **(C)**. **(D)** The differential co-expression network. Every node represents a gene, the inner part of the node is coloured according to its 4-day module, while the outer rim is coloured according to its 8-day module. Gene-pairs can be: positively correlated in both exposure periods (Conserved + ve), positively correlated in one period and negative in the other (Differentiated) or correlated only in 4 days exposure (Specific_4d) or only in 8 days (Specific_8d). The NCBI gene IDs for the focused DiCE genes are listed in Supplementary Table S4.

Our modules revealed that short- and long-term exposure affect the regulation of many of the same pathways, but with the long-term exposure activating a wider range of pathways. We wanted to compare the co-expression structures (modules) discovered in the 4- and 8-days data to identify similarities and dissimilarities. To this end, we performed a differential co-expression (DiCE) analysis. All gene-pairs were classified into three categories (or otherwise remained unclassified): co-expressed at both exposure periods (conserved co-expression, Figure 4A), co-expressed only at one period (specific co-expression, Figure 4B) or positively co-expressed in one period and negatively co-expressed in the other (differentiated co-expression, Figure 4C). We then identified hubs in the differential network constructed by connecting gene-pairs with co-expression classified into one of these three categories. In total, this network contained 609 genes with 5417 conserved connection, 1100 specific connections and 138 differentiated connections (Figure 4D).

The differential network is dominated by genes with conserved co-expression in both exposure periods reflecting the similarities in our functional characterization of the modules. The different conserved subnetworks also highlight module-pairs from the two exposure periods with overlapping gene content. Indeed, a detailed analysis of all module-pairs revealed that 22 of the 38 4-day modules (58%) and 22 of the 36 8-day modules (61%) overlapped strongly (p < 1e-5) between the two exposure periods (Supplementary Figure S7A). An example of a gene that is co-expressed with many genes in both exposure periods (i.e., conserved hub) is the putative cuticular protein (LOC116920760), which belongs to the **turquoise.4d** and the **brown.8d** (Figure 4). This gene is central in the high degree of overlap observed between these two modules. The importance of this gene in response to environmental perturbations and predator cues was previously demonstrated in insects such as *D. melanogaster* and *Daphnia* spp (Orsini et al., 2018; Brown et al., 2014).

To understand the differences in transcriptional response between the two exposure periods, we examined hub genes that were specifically or differentially co-expressed in the differential co-expression network. These included 133 genes co-expressed only after 4 days (Supplementary Figure S7B) and 196 genes only co-expressed after 8 days exposure to radiation (Supplementary Figure S7C). Although these genes belong to a wide variety of modules, they share the property that their co-expression pattern changed between 4 days and 8 days. Interestingly, these genes were found to largely be enriched in the same functional categories (Supplementary Figure S8), thus showing that the two exposure periods activated the same processes through different genes.

One subnetwork of the differential network is dominated by genes specifically co-expressed in the 8 days data. While most of these genes reside in the **turquoise.8d** module, they belong to a myriad of 4 days-modules. These genes are enriched for processes related to the formation of heme, crucial for producing haemoglobin (Supplementary Figures S8C, D). One example is the two highly interconnected di (heme)-domain haemoglobin genes (*Dhb1*), which specifically co-expressed with over 60 genes. Another example is vitellogenin (*Vtg2*), which belong to the **grey.4d** and two vitellogenin fused superoxide dismutase proteins, which associated with reproductive strategies. Interestingly, these two VTG-SOD genes are assigned to different modules: **red.8d** containing the *Br* TF and putative homolog of ecdysteroid regulated 16 kDa protein (ESR16), and **yellow.8d** containing SOD1. In *Bombyx mori, Br* is crucial for the oocyte formation and regulates the TFs of vitellogenin (Nojima et al., 2019), whereas ESR16, downregulated by ecdysteroid (a regulator of *Vtg2*) thereby triggering pupal diapause through lipid metabolism (Liu et al., 2023). Collectively, these specifically co-expressed genes may implicitly link the underlying cellular ROS defence mechanism to accelerated oocyte maturation.

### Multi-omics integration of transcriptomics and metabolomics data

**FIGURE 5:**
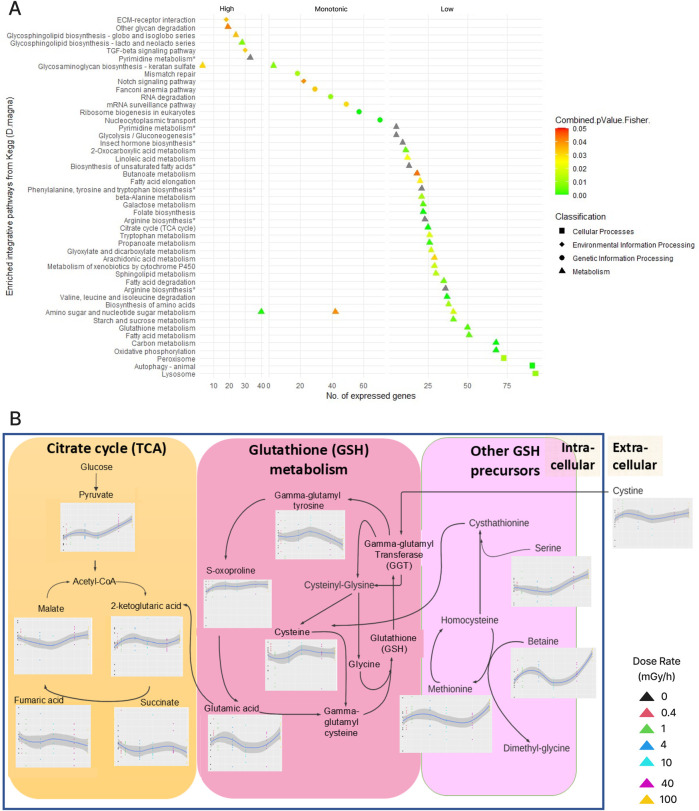
**(A)** 50 enriched KEGG pathways grouped according to the differential abundance type: high dose-rate responsive genes/metabolites, monotonically increasing/decreasing or low dose-rate responsive. Pathway with “*” did not pass the combined *p* < 0.05 cutoff but were enriched in either the transcriptomics or metabolomics data. **(B)** Dose-rate responsive metabolites in pathways related to reprogramming of energy metabolism during the increase of intracellular ROS: Tricarboxylic acid (TCA) cycle, glutathione (GSH) metabolism, and other GSH precursors. Detailed biological interpretation of the integrated multi-omics pathways **(A)**, **(B)** along with differential metabolites were documented in the supplements.

To gain further insights into the biological consequences of the transcriptional responses to radiation, we next integrated the transcriptomics data with metabolomics data from an 8-days exposure experiment (Figure 5). Of 195 identified and quantified metabolites, we detected differential metabolites (DM) based on the dose rates responses: 123 metabolites with a monotonic increase or decrease in abundance, 51 metabolites with low dose-rate response (0 vs. 1 mGy/h) and 93 metabolites with high dose-rate response (1 vs. 100 mGy/h) (Supplementary Figure S9). Using these differential metabolites as well as genes from the differential co-expression network and the significant modules, we identified 133 pathways of which 50 pathways were significantly enriched: 34 were associated with low dose-rate responses, 7 with high dose responses and 9 with monotonic increases/decreases (Figure 5A), revealing enrichment multi-omics pathways highly relevant to our DiCE network.

Pathways with a monotonic response to radiation were dominated by developmental signalling (Notch signalling pathway) pathways related to genetic information processing such as protein translations, mRNA syntheses and DNA repair, as well as transport between the nuclei and the cytoplasm. The pathways “glycosaminoglycan (keratan sulphate) biosynthesis” (high and monotonic) and “amino sugar and nucleotide sugar metabolism” (high, monotonic and low) were linked to multiple dose responses. Keratan sulphate typed glycosaminoglycan are components of the ECM synthesised by the ER and Golgi in the central nervous system, it plays a vital role in developmental and glial scar formation after tissue injury (Zhang et al., 2006). Amino sugar and nucleotide sugar metabolism are essential for chitin and chitosan structure, with derivatives exhibiting free-radical scavenging, neuroprotective and anti-inflammation characteristics as part of the innate immune system of crustacean against oxidative damage.

Pathways associated with low dose-rate response (0–1 mGy/h) were dominated by metabolic pathways related to energy homeostasis. A surge of pyruvate (Figure 5B) and the accumulation of OXPHOS intermediate substrates (elevated NADH/NAD+ and FADH2/FAD) were observed, indicating mitochondrial uptake inhibition and a shift towards anaerobic and aerobic glycolysis (Warburg effect) rather than oxidative phosphorylation (OXPHOS) (Yang et al., 2014; Liemburg-Apers et al., 2015; Navas and Carnero, 2021). The enrichment of the glutathione (GSH) synthesis pathway in carbon metabolism was enriched, mediating repair mechanisms and promoting cell survival upon ionizing radiation exposure (Estrela et al., 2006; Pujari et al., 2009). However, the non-monotonic elevation of GSH precursors (Figure 5B), suggests that the enrichment of fatty acid metabolism triggered by accumulating GSH as another major source of energy to replace glucose, promoting fatty acid beta oxidation (Aledo, 2004).

High dose-rate responsive pathways were associated with the remodelling of cell-bound factors within the extracellular matrix (ECM), substrate recycling, and redox signalling (modification of glycosphingolipid and glycosaminoglycan biosynthesis at the cell membrane). Additionally, the enriched TGF-beta related signalling pathways indicated alterations in gene expression of ECM synthesis and degradation, cell differentiation during embryogenesis, and immune system signalling during cellular injury and healing (Patterson and Padgett, 2000; Verrecchia and Mauviel, 2002).

Taken together, different dose-response patterns in genes and metabolites revealed distinct pathways and mechanisms of radiation response in *D. magna*, suggesting a method to dissect this response into more manageable components (see Discussion).

## Discussion

In this study, we developed a semi-supervised workflow to analyse dose-response patterns in multi-omics data and demonstrated its utility on a previously published dataset from *D. magna* exposed to gamma radiation. The workflow integrates several methods that, although not novel in themselves, were shown to generate novel insights. For example, our workflow was able to predict that reproduction-relevant delay was likely caused by disruption in haematopoiesis. This was not reported in the previous studies, but was consistent with their bioassays.

Although co-expression network analysis is unsupervised by nature (i.e., discovers natural groups – modules – in data with no prior knowledge of these groups), the workflow is semi-supervised because it facilitates the use of prior knowledge about radiation responses to select relevant modules (in this study: low and high dose-rate-responsive genes as well as genes with a monotonic response). Thus, the approach starts out by performing an explorative analysis (unsupervised), but then allows the user to perform targeted module selection (supervised). This supervised step is not mandatory and can be bypassed to keep the workflow purely exploratory. The modules are also characterised using gene function enrichment and transcriptional regulators are predicted using transcription factor motif enrichment analysis. Finally, pathway analysis is used to integrate metabolomics data. Taken together, this workflow allowed us to leverage expression patterns, metabolites, gene function, pathway information, and regulators to dissect the molecular radiation responses of *D. magna*. To our knowledge, no other method in the field of toxicology assessment combines these elements in one user-friendly workflow.

The workflow also includes an implementation of differential co-expression network analysis (DiCE). This method identifies genes (hubs) that change their co-expression relationships with many other genes between data sets and can therefore be used to compare different experiments. In this study, we used differential network analysis to systematically compare the modules from short- and long-term exposure (4- and 8-days) to identify genes that were assigned to a module in one exposure period but not in the other. For example, the **turquoise.8d** module contained several hub genes specifically co-expressed in the 8-days exposure data (Figure 4). These genes included key players of mitochondrial heme (porphyrin) production, such as uroporphyrinogen decarboxylase (HemE) and coproporphyrinogen oxidase (HemF) (Ogun et al., 2019) as well as two di (heme)-domain haemoglobin genes (*Dhb1*) (Supplementary Figure S10I: 4-days and 8-days). Multiple studies have shown an increase in the mRNA levels of *Dhb1* in *D. magna* as an adaptation to adverse environments, promoting high oxygen affinity and supply to tissues and organs (Kato et al., 2001; Gorr et al., 2004; Gerke et al., 2011). However, dysregulation of mitochondrial heme production may result in an excess of intracellular heme. Free heme has been associated with various toxic effects including lysis (cell death) through lipid peroxidation (Chiabrando et al., 2018). The specific co-expression of *HemE*, *HemF* and *Dhb1* in adult *D. magna* (8-days), but not in juvenile individuals (4 days), indicate adult-specific regulation of heme availability during radiation exposure. One candidate for this adult-specific regulation of heme availability is the haemocytes, which are responsible for the precursor uptake in the heme biosynthetic pathway (Fredrick and Ravichandran, 2012).

Differential co-expression networks may also be used to identify genes that respond robustly to radiation (biomarkers). Indeed, previously discovered biomarkers such as *Sod, Nat,* and *Vtg* (Song et al., 2020) were co-expressed with many other genes in both exposure periods in our differential network (conserved hubs). Hence, conserved hubs allow us to identify potentially novel biomarkers with similar putative functions. Some such examples include DNA directed RNA polymerase II *Rbp1* subunits, glucose dehydrogenase FAD, cAMP-dependent protein kinase catalytic subunit (*Campk*), sodium/potassium-transporting ATPase subunit beta subunit 1 (*Atp1b1*) and chorion peroxidase. (Kato et al., 2001; Gorr et al., 2004; Gerke et al., 2011; Chiabrando et al., 2018; Fredrick and Ravichandran, 2012; Song et al., 2020; Viant et al., 2019). The distinct radiation response mechanisms revealed by multi-omics pathway analysis of different dose-response patterns suggested using our workflow to first select pathways (Figure 5A), and then interpret these by leveraging modules and module annotation, differential co-expression and the regulatory network. Below, we provide examples demonstrating how this method can yield new insights into the gamma radiation response in *D. magna*.

### Monotonic response linked to genetic information processing

The Notch signalling pathway was enriched in genes and metabolites with a monotonic dose rate response in the multi-omics pathway analysis (Figures 5A, Supplementary Figure S10B). This pathway is known for its diverse roles in cell differentiation, ecdysteroid production, moulting and ROS homeostasis, and has also been reported to promote radio-resistance under hypoxic conditions, sharing signalling pathways with both intrinsic (initiated by mitochondrial membrane receptors) and extrinsic (initiated by plasma membrane receptors) apoptotic factors (Yahyanejad et al., 2016). As the over-represented multi-omics pathways, which include protein translations (ribosomal biosynthesis in eukaryotes), mRNA syntheses (mRNA surveillance pathway, RNA degradation) and DNA repair mechanisms (Fanconi Anaemia pathway, mismatch repair), are similar to the module enrichment analysis (Supplementary Figure S10N). This suggests that the interplay between these pathways is likely controlled by Notch signalling.

In the 4-day regulatory networks (Figure 3), the downregulation of the TF homolog HHEX from the monotonic blue module may indicate decreased signalling activity of Rho GTPase cycle. The downregulation of TF homolog *HHEX* from the **blue.4d** module of regulatory network (Figure 3) has been previously implicated in contributing to early stages of diapause phenotypes, including arrested cell cycle, development, and an increase in metabolic reserve in alfalfa leaf cutting bees (Yocum et al., 2018). These findings coincide with observations from a previous study (Song et al., 2020), in which an altered reproductive strategy shifted from a delayed (4 days) to an accelerated reproduction cycle (8 days). In crustaceans, the haematopoietic system is located close to the vascular system to transport nutrients, hormones, and immune cells throughout the body. The blood progenitor cells of *Drosophila* are reported to be sensitive to internal and external stress, coordinating the developmental pathways throughout the life cycle with the activation of Notch signalling (Banerjee et al., 2019).

Collectively, the monotonic responsive group suggests that radiation affects the genetic processing and altering the growth with Notch signalling as the modulator.

### Low dose rates responses are linked to oxidative damage in energy homeostasis

Notch signalling can orchestrate a metabolic switch from “normal” TCA and oxidative phosphorylation to anaerobic glycolysis, thus increasing radiation resistance by reducing intrinsic and extrinsic ROS (Yahyanejad et al., 2016). Interestingly, the glycolysis pathway was found enriched in genes and metabolites responding to low dose rate radiation (0 vs. 1 mGy/h) (Figure 5A). Levels of pyruvate (an intermediate in glycolysis) significantly increased in low dose-rate irradiated cells (Figure 5B) and, given that anaerobic glycolysis takes place in the cytosol and not the mitochondria, this could suggest reduced mitochondrial uptake of pyruvate as the organism switches to anaerobic glycolysis (Supplementary Figure S10M). We also found that glutathione (GSH) and fatty acid metabolism related pathways responded to low dose radiation (Figures 5A, Supplementary Figure S10C). These are linked to amino acid metabolism and immune response that counterbalance damages from cellular ROS, and are part of an energy compensation strategy to support TCA when glucose-derived carbon is highly demanded (Yang et al., 2014; Navas and Carnero, 2021). Indeed, we found elevated abundances of α-ketoglutaric acid and glutamic acid at low dose rates (Figure 5B). Moreover, the GSH precursors (glutamine, cysteine, methionine, betaine, 2-oxoproline, and glycine) are amino acids well known for modulating crustacean innate immunity (Huang et al., 2020). However, insufficient GSH due to deprivation of cysteine can trigger ferroptosis, supporting observations include DiCE genes *Dhb1* as disturbed iron homeostasis, glutathione state, amino sugar and nucleotide sugar metabolism (Supplementary Figures S10E, D, Q: enriched TCA and elevated OXPHOS substrate NAD+), and lipid peroxidation in metabolic pathways (Hao et al., 2018). Despite no metabolite trace of GSH being found, the enriched pathway (Supplementary Figure S10G) and constituents suggest an increase in GSH synthesis is one of the early defences induced by ionizing radiation (Pujari et al., 2009, Kim et al., 2003; Wang et al., 1997). The reprogramming of energy metabolism suggested by the multi-omics pathway analysis might fuel the radio-resistance activities captured by the network modules in DNA repair, oxidative stress relief and autophagy (Supplementary Figure S10L). Indeed, several low dose rate responsive modules (**brown.4d**, **yellow.4d**, **turquoise.4d**, and **pink.4d**) were enriched for the pathways “RHOA GTPase cycle” and “signalling by Rho GTPases”, which are known to be associated with actin cytoskeletal reorganisation and regulation of highly proliferating cells.

Taken together, low dose response is associated with a reprogramming of the energy metabolism to combat increased oxidative stress.

### High dose rate response linked to cell membranes and cell signalling

The multi-omics pathway analysis “glycosaminoglycan (keratan sulphate) biosynthesis” (high dose and monotonic) orchestrated by the central nervous system (CNV), is responsible for the synthesis of ECM components created by the ER and Golgi (Supplementary Figure S10P). Similarly, amino sugar and nucleotide sugar serve as essential structural components of chitin and chitosan, indicating ECM structural modification (Supplementary Figures S10H, Q). While limited studies linking the effect of radiation with the enriched “Glycosphingolipids (GSL) biosynthesis,” GSL carried the hydrophilic glycan epitopes (produced in ER and Golgi) was responsible for cell surface protein interaction (Satake and Miyamoto, 2012). Concurrently, significant decrease in glucose (−0.4 fold, *p* < 0.012), fructose (−0.08, p-val <0.003) and verbascose (−0.5 fold, *p* < 5.4E-10) may also indicate signals of substrate recycling of glycosaminoglycan and glycan (Figure 5A) in the ECM to achieve energy homeostasis under high oxidative stress. Other enriched pathways include the enriched of pyrimidine metabolism (increase UMP-glucose, *p* < 0.002, Supplementary Figure S10J: 8-days) to supply GSL production, TGF-beta related signalling pathway (Supplementary Figure S10F) which controls the signalling during ECM synthesis and degradation, and the ECM-receptor interaction (Patterson and Padgett, 2000; Verrecchia and Mauviel, 2002). Taken together, the pathways identified by multi-omics integration analysis suggested that the physiological membrane concentrations facilitate the crosstalk with ROS through redox signalling, orchestrated by the central nervous system.

Module based pathway enrichment shows that “Golgi to ER retro-trafficking,” “ER to Golgi Anterograde Transport,” “COPI-mediated anterograde transport,” and “COPI-dependent Golgi-to-ER retrograde trafficking,” along with “O-linked glycosylation” (occurs in Golgi) and “N-glycosylation” (occurs in ER) are highly enriched in the **darkred**, **salmon**, and **yellow** modules at 4-day data. Shuttle protein METTL between ER and Golgi belongs to the methyltransferase-like protein family, a highly conserved DiCE hub gene in two exposure periods (**purple.4d**, **pink.8d**), was regulated by RhoBTB activity which can be found in significant modules (**black.8d**, **red.8d**, **lightyellow.8d** and **salmon.8d**) that overlapped in both monotonic response and the high dose-rates responsive group (McKinnon and Mellor, 2017; Qi et al., 2023). A previous study reported that the METTL protein family is involved in the RNA modification in *Metazoa* and performs a variety of epigenetic functions (Wong and Eirin-Lopez, 2021).

These pathways are known for tracking down misfolded and misassembled protein from ER to Golgi before glycosylation or disulfide bond formation (Perez-Linero and Muñiz, 2015). However, faulty proteins might escape the ER to get to the Golgi. The escaped proteins are tracked down by coat protein I (COPI) in Golgi and shipped back to ER through retrograde transport vesicles (Perez-Linero and Muñiz, 2015). Interestingly, these modules tend to increase in expression for medium doses (4–40 mGy/h) but then drop in expression for the highest dose (100 mGy/h). This observation suggests that protein tracking is triggered by specific dose rates, but that the emphasis of the cellular defence shifts towards other responses at very high dose rates.

Noteworthy, while the “O-linked glycosylation” identified through DiCE analysis in both 4-days and 8-days exposure (Supplementary Figure S10), is highly relevant to the functionalities of these multi-omics pathways in triggering ECM and nucleic acid remodelling through cell signalling (Supplementary Figures S10O, Q), it also frequently appears among the high dose rate responsive modules in both radiation exposures (4d: **darkred**, **salmon**, **yellow**, **turquoise**, **green**, **tan**; 8d: **brown**, **greenyellow**, **lightcyan**, **magenta**, **red**, **darkturquoise**). This pathway presents in various cellular compartments (ER, Golgi apparatus, ECM, and cytoplasm) of eukaryotes, was reported to be involved in diverse protein modifications, including the O-linking of different glycans to serine or threonine through mucin core-2 during glycosylation (Kusche-Gullberg and Kjellén, 2003). In *Drosophila*, mRNA of O-linked glycans, particularly mucin type (>90%), with galactosyltransferase activity, is detected in various tissues, suggesting its significance in neural development (Lin et al., 2008). Recent findings indicate the attachment of extracellular O-linked glycan (O-GlcNAc) to membrane proteins, particularly epidermal growth factor (EGF) repeats. This evolutionarily conserved motif plays a role in down-regulating Notch signalling, influencing pyrimidine metabolism (Zhang and Ten Hagen, 2019). O-GlcNAc addition to O-fucose on Notch receptors triggers *fringe*, a glycosyltransferase controlling cellular communication by binding to uracil-diphosphate glucose (UDP). These are thus examples of the two exposure periods activating the same processes triggered by high dose-rate of radiation through different genes.

In the 8-days TF regulatory network, several high dose rate responsive modules predicted to regulate the metabolism of macromolecules and the organ system (**black** and **green**), as well as the signalling pathway in transmitting the messages of alteration (**salmon**). Interestingly, the frequent occurrences of Br TF homologs (Figure 3B) function as repressors of the **blue** module at high dose rates, was reported induced by ecdysone, controlling the hormonal crosstalk which influences metamorphosis, morphogenesis, and ovarian development (Jiang et al., 2017). Recent research indicates the interplay between ecdysteroids and juvenile hormones can induce haemoglobin-related genes in *D. magna* while also activating the male sex determining genes during oocyte maturation (Gorr et al., 2006). A suppression in the transcriptional expression of VTG-SOD and isoforms as these physiological responses were connected by the same juvenoid pathway. Another study demonstrated the disturbance in mRNA level of the juvenile hormones and ecdysteroid regulated protein impairs the transfer of triacylglycerols into the egg yolk (Jordão et al., 2016). This results in increased lipid storage in the fat cells of postspawning adult females, consequently reducing the fitness of maternal daphnids and their offspring (Jordão et al., 2016). These findings seem aligned with the DiCE pathway enrichment output (heme metabolism, O-linked glycosylation), GO term (structural constituent of cuticle, lipid transporter activity, response to oxidative stress and etc.) and specifically co-expressed genes (*Esr16, Sod*, *Vtg2*, *Vtg-Sod*, *hemEF* and *Dhb*) from the 8 days-DiCE network.

The involvement of O-linked glycosylation-related signalling, which interacts with the Notch signalling pathways and ecdysteroid-regulated genes, alongside haemoglobin formation within the 8-day timeframe, collectively implies that disruptions in haematopoiesis contribute to the disturbance of sexual maturation, egg production, and the endocrine system resulting from prolonged radiation exposure.

## Data Availability

The raw data supporting the conclusions of this article will be made available by the authors, without undue reservation.

## Abbreviations

SOLA, Semi-supervised Landscapes Analysis; MOA, Mode of Action; POD, Point of Departure; AOP, Adverse outcome pathways; WGCNA, Weighted Gene Co-expression Analysis; GO, Gene Ontology; DiCE, Differential Co-expresssion Analysis; DEGs, Differentially expressed genes; DMs, Differential metabolites; ReactomePA, Reactome Pathway Enrichment Analysis; ROS, Reactive oxygen species; TFBS, Transcription factor binding sites; CNV, Central Nervous System; ECM, Extracellular matrix; GSH, Glutathione; O-GlcNAc, O-linked glycan.
