# Supplementary material for: SOLA: dissecting dose-response patterns in multi-omics data using a semi-supervised workflow
Source: Front Genet. 2024 Dec 2;15:1508521. doi: 10.3389/fgene.2024.1508521 (PMC11647027; doi:10.3389/fgene.2024.1508521)
Supplement: Supplementary file 1 [file DataSheet1.zip › Supporting information/Functional_enrichment_4d.html]

Functional enrichment analysis


# Functional enrichment analysis

#### Wanxin Lai

#### 13/03/2022

```
library(tidyverse)
library(dplyr)
library(GeneOverlap)
library(DOSE)
library(clusterProfiler)
library(ReactomePA)
library(enrichplot)
options(connectionObserver = NULL)
library(org.Dm.eg.db)
library(ggnewscale)
library(data.table)
```

```
dir.create(paste("../07_Functional_Enrichment/LOCidmodule-", params$exposure, "ays/", sep = ""))

dir.create(paste("../07_Functional_Enrichment/LOCidmodule-", params$exposure, "ays/Functional_Enrichment", sep = ""))
```

### Reactome pathway over-representation analysis

```
modulesgenes <- list.files(paste("../06_Identifiers_Conversion/degsEntrez-", params$exposure, "ays/", sep =""), pattern= paste(".*\\-sigModEntrez-", params$exposure, "ays.txt$", sep =""), full.names=TRUE)

list.of.module.frames <- lapply(modulesgenes, read.table)
```

```
for(i in 1:length(list.of.module.frames)){
  
  if(nrow(list.of.module.frames[[i]]) > 10) {
    
    de <- as.character(list.of.module.frames[[i]]$V1)
    
    colour.of.module <- substr(modulesgenes[i], start = 47, stop = (nchar(modulesgenes[i])- 23))
    
      
    test <- enrichPathway(gene = de, pvalueCutoff = 0.05, organism = "fly", pAdjustMethod = "none", minGSSize = 10)
    
    test@result$GeneRatio <- parse_ratio(test@result$GeneRatio)
    test@result <- test@result[order(test@result$GeneRatio, decreasing = TRUE),]

    write.csv(test@result, file = paste("../07_Functional_Enrichment/LOCidmodule-", params$exposure, "ays/Functional_Enrichment/", colour.of.module,"-",length(de),"React-PA.csv", sep = ""), row.names = FALSE)
    
    test_top <- test@result[1:20,]
    
    
    p <- test_top %>% ggplot(aes(x = GeneRatio, y = reorder(stringr::str_wrap(Description, 70), dplyr::desc(as.numeric(pvalue))), size = Count, colour = pvalue))
    p <- p + geom_point()
    p <- p + scale_colour_gradientn(limits = c(0, 0.05), #max(test_top$pvalue)),
  colours=c("blue", "yellow", "red"))
    p <- p + ggtitle(paste("Module:", colour.of.module))
    p <- p + ylab("")
    
    print(p)
  }
}
```

### GO analysis

```
#import GOterm file
term2name <- read.table("../lookup-db/goterms.txt", header = TRUE)

# import dme's annotated GO file
Gene.GOid <- read.table("../lookup-db/XM-GO.dbres.txt")
term2gene <- data.frame(Gene.GOid$V2,Gene.GOid$V1)

sigmodule <- read.table(paste("../05_Module_Selection/sigmod-all-", params$exposure, "ays.txt", sep =""))[,"V1"]
```

```
#*******This time we fill modules with XM ids for GO analysis*****#

inXMmodules <- vector()

for(i in sigmodule){
  
  #load module genes
 inXMmodules <- append(inXMmodules, paste("../03_WGCNA/WGCNA-", params$exposure, "ays/Hubs05-",params$exposure, "aysBWnet/", i,"-HUBs-", params$exposure, "ays-namelistBW.txt", sep = ""))
}

list_of_module_frames <- lapply(inXMmodules, read.table)


for(i in 1:length(list_of_module_frames)){

  # nrow must be greater than 10 for enricher to work
  if(nrow(list_of_module_frames[[i]]) > 10) {
    
    module_colour <- list_of_module_frames[[i]]$group[1]
    gene <- as.character(rownames(list_of_module_frames[[i]])) 
    #gene <- as.character(list_of_module_frames[[i]]$V1)
    
    #universal enrichemnt analysis
    unigo <- enricher(gene,TERM2GENE = term2gene, TERM2NAME = term2name, pvalueCutoff = 0.05, pAdjustMethod = "none", qvalueCutoff = 0.1)
    
    #parse ratio convert strings into decimals
    unigo@result$GeneRatio <- parse_ratio(unigo@result$GeneRatio)
    unigo@result <- unigo@result[order(unigo@result$GeneRatio, decreasing = TRUE),]
    
    write.csv(unigo@result, file = paste("./LOCidmodule-", params$exposure, "ays/Functional_Enrichment/",module_colour,"-",length(gene),"GO.csv", sep = ""), row.names = FALSE)
     
    unigo_top <- unigo@result[1:20,]
    
    p <- unigo_top %>% ggplot(aes(x = GeneRatio, y = reorder(stringr::str_wrap(Description, 50), dplyr::desc(pvalue)), fill = pvalue))
    p <- p + geom_col()
    p <- p + ggtitle(paste("Module:", module_colour))
    p <- p + ylab("")
    
    #ggsave(paste(module_colour,"-bw8d-GOenrich.pdf"))
    print(p)
    print(paste("Number of genes in ",module_colour, "module:",length(gene)))
    
  }
}
```

```
## [1] "Number of genes in  blue module: 363"
```

```
## [1] "Number of genes in  darkred module: 101"
```

```
## [1] "Number of genes in  pink module: 266"
```

```
## [1] "Number of genes in  salmon module: 136"
```

```
## [1] "Number of genes in  turquoise module: 541"
```

```
## [1] "Number of genes in  brown module: 316"
```

```
## [1] "Number of genes in  green module: 199"
```

```
## [1] "Number of genes in  yellow module: 263"
```

```
## [1] "Number of genes in  tan module: 202"
```
