# Supplementary material for: SOLA: dissecting dose-response patterns in multi-omics data using a semi-supervised workflow
Source: Front Genet. 2024 Dec 2;15:1508521. doi: 10.3389/fgene.2024.1508521 (PMC11647027; doi:10.3389/fgene.2024.1508521)
Supplement: Supplementary file 1 [file DataSheet1.zip › Supporting information/Supplementary Materials.pdf]

Supplementary Material

Article title:

SOLA: Dissecting dose-response patterns in multi-omics data using a semi-supervised workflow

Authors: Wanxin Lai, You Song, Knut-Erik Tollefsen, Torgeir R. Hvidsten

Contents

Fig. S1: Overview of software in each step of data analysis. R commands are shown in italic. ... 4

Fig. S2: WGCNA. Diagnostic plots showing various beta fits to reach a scale-free topology network. Analysis of scale-free network topology using different soft-thresholding power on 4 days transcriptomics data. A and B show the numbers of genes in every module, which was given an arbitrary colour. C and D show the scale free fit index (y-axis) whereas E and F show the mean connectivity (degree, y-axis). 5
Fig. S3: (A) Volcano plots showing DEGs from linear combinations (DESeq2). (B) Examples of the eigengene expression profiles showing monotonic (top), and non-monotonic: low-(middle) and high (bottom) dose rates responses. Eigengene module is the first principal component which best-represents the gene expression profile of a module. 7
Fig. S4: All significant modules in the 4-days (A) and 8-days (B) gamma radiation exposure data. Each module is represented by its eigengene (the first principal component of the module); y-axis showing the expression (Log2FC), x-axis showing the dose rates. 8
Fig. S5: Significant modules detected through the overlapped of WGCNA and DESeq2 though the Fisher' s Exact test. The number of DEGs in modules from 4 days (A) and 8 days (B) of radiation exposure were showed by the y-axis and the name of modules were indicated by the x-axis. Three types of DEGs were identified: monotonic increase/decrease (termed "Linear" for simplicity), low dose responsive (Low) and high dose responsive (High). The thick black borders mark modules that are significantly enriched in DEGs. 9
Fig. S6: Venn diagram shows number of significantly enriched GO terms for 4 days vs 8 days radiation exposure (A-B) and the enriched Reactome pathway (p-value < 0.05) (C-D). The six Venn diagrams above depict the number of genes that were involved in the Reactome overrepresented pathway analysis (A) and the number of pathway dedicated to each exposure period (B). The top enriched Reactome pathway are labelled by different exposure periods. The six Venn diagrams below are the output from Reactome PA, the number of genes involved in GO analysis (C) and the labels indicate the top enriched pathways from each period (D). 14
Fig. S7: (A) Statistically significant overlap in gene content between modules discovered in the 4 days and 8 days data. Modules with significant changes in expression are marked with an asterisk (*). (B-C) The centrality (number of connections) of genes in the DiCE network grouped by module: 4 days (left) and 8 days (right). Genes can be: positively correlated in both exposure periods (Conserved +ve), positively correlated in one period and negative in the other (Differentiated) or correlated only in 4 days exposure (specific_4d) or only in 8 days (specific_8d). 15
Fig. S8: Functional annotation of DICE selected genes from 4 and 8 days with GO enrichment analysis (top A and B) and Reactome PA (bottom C and D). 16
Fig. S9: Plots show the initial analysis on metabolites abundance. (A) PCA (Principal Component Analysis) plot showed the sample of metabolites in 2D plane spanned by the first two principal components which explained the most variance. No clustering pattern observed indicates an extremely small difference between samples. (B) Venn diagram comparing the numbers of DEMs shared and uniquely existing between low dose-responsive, high-dose responsive and linear model groups. (C) Heatmap showing the gene expression of all metabolites and all samples; red to blue colour scale represents high to low gene expression and the colour of dose rates was represented by the legend on the right. 17
***Fig. S10:** Paintomics integrated pathway enrichment analysis* 18

Fig. S11: Comparison of orthologous genes between different clones of Daphnia magna, Daphnia pulex and Drosophila melanogaster. A: Venn diagram showing the numbers of shared orthologous groups between D. pulex, D. magna xinb3, D. magna KIT and D. melanogaster. B: The bar graph above shows the numbers of protein clusters found in each species, while the bar plot below displays the number of orthologous clusters shared by 1, 2, 3 and 4 species. C: Pairwise heatmap with number of overlapping clusters between different pairs of species. The overlapping cluster numbers were indicated in the cells and the colour intensity followed the shared number of orthologous groups: the darker the colour, the more orthologs shared between species. 29

Table S1 The number of differentially expressed genes generated by DESeq2 in three different design group(linear, low-, and high- dose rate responsive group). Except for Ctrl vs 1 which is a low dose rate-responsive group, the data from 8 days has more DEGs than from 4 days in the high dose-rate responsive group (1 vs 100) and the linear model. 5

Table S2: TF (transcription factors) orthologs and their corresponding modules from the data of 4 days of gamma radiation exposure. Gene symbols and gene names followed the nomenclatures of D. melanogaster as documented in Flybase. TFs (transcription factors) are considered activated if a corresponding enriched motif was found and the ortholog genes which encoded for the TFs were present in that module. 10

Table S3: TF orthologs and their corresponding modules from the data of 8 days of gamma radiation exposure. Gene symbols and gene names followed the nomenclatures of D. melanogaster as documented in Flybase. TFs are considered activated if a corresponding enriched motif was found and the ortholog genes which encoded for the TFs were present in that module. 11

Table S4: DiCE genes and their corresponding NCBI gene identification. 28

Steps of data analysis	Overview of software
Data pre-processing	DESeq2<br>• downloaded from Bioconductor<br>• *vst* transformation<br><br>PCA<br>• from package 'stats' included in R<br>• *prcomp* for computing the PC (Principal Component)
Identification of modules and DEGs	DESeq2<br>• *DESeqDataSetFromMatrix*<br>• *deseq* for DE analysis<br><br>WGCNA<br>• downloaded from Bioconductor<br>• *DeseqDataSetFromMatrix* for creating object that store input and intermediate values<br>• *blockwiseModules* to identify modules<br>• *factor* to create design matrix.
Identification and detection of biologically meaningful modules	GeneOverlapp<br>• downloaded from Bioconductor<br>• *newGeneOverlap* for Fisher exact test
Conversion of identifiers	BLAST2GO<br>• paid commercial software<br>• available for Windows system (7+)<br>• Refseq -> GO ID<br>• Refseq -> Entrez ID<br><br>ncbi dataset<br>• command line tool<br>• run in Linux system<br>• mrna sequence -> gene sequence<br><br>OrthoVenn2<br>• online platform for othologous gene comparisons using DIAMOND<br>• Using OrthoMCL for gene clusterings<br><br>KAAS<br>• online plarform<br>• based on KEGG database<br>• Refseq -> KO ID
Detection of biologically meaningful modules and construction of transcriptional regulatory network	ReactomePA<br>• downloaded from Bioconductor<br>• *enrichPathway* for identifying enriched pathway from modules<br><br>clusterprofiler<br>• downloaded from Bioconductor<br>• *enricher* for GO overrepresentation analysis from modules<br><br>Bedops<br>• command line tool downloaded from github<br>• run in Linux system<br>• extract upstream gene sequence<br><br>AME<br>• command line tool for motif enrichment analysis<br>• run in Linux system<br><br>OrthoFinders<br>• command line tool<br>• software downloaded from github<br>• detect orthologous sequence using DIAMOND with 'ultra-sensitive' mode<br><br>NCBI Datasets<br>• web service maintained by NCBI<br>• useful in downloading small numbers of protein sequence
Integration of metabolomics and transcriptomics data	limma<br>• downloaded from Bioconductor<br>• *lmFit* for estimating the logFC<br>• *empiricalBayes* for estimating standard error<br>• *makecontrast* for contrasting dose rates of interest<br>• *contrast.fit* for final coefficients and standard errors<br>• *topTable* for displaying results<br><br>BiNGO<br>• Cytoscape plugin<br>• GO analysis for DEACGs<br><br>AutoAnnotate<br>• Cytoscape plugin<br>• Assigned new annotations for similar GO clusters<br><br>EnrichmentMap<br>• Cytoscape plugin<br>• visualization of enriched GO and corresponding cluster of genes<br><br>Paintomics<br>• online platform<br>• integrating the DEACGs and DEMs for enriched pathway / chemical reaction analysis<br><br>ReactomePA<br>• *enrichPathway* for enriched pathway analysis for DEACGs

Fig. S1: *Overview of software in each step of data analysis. R commands are shown in italic.*

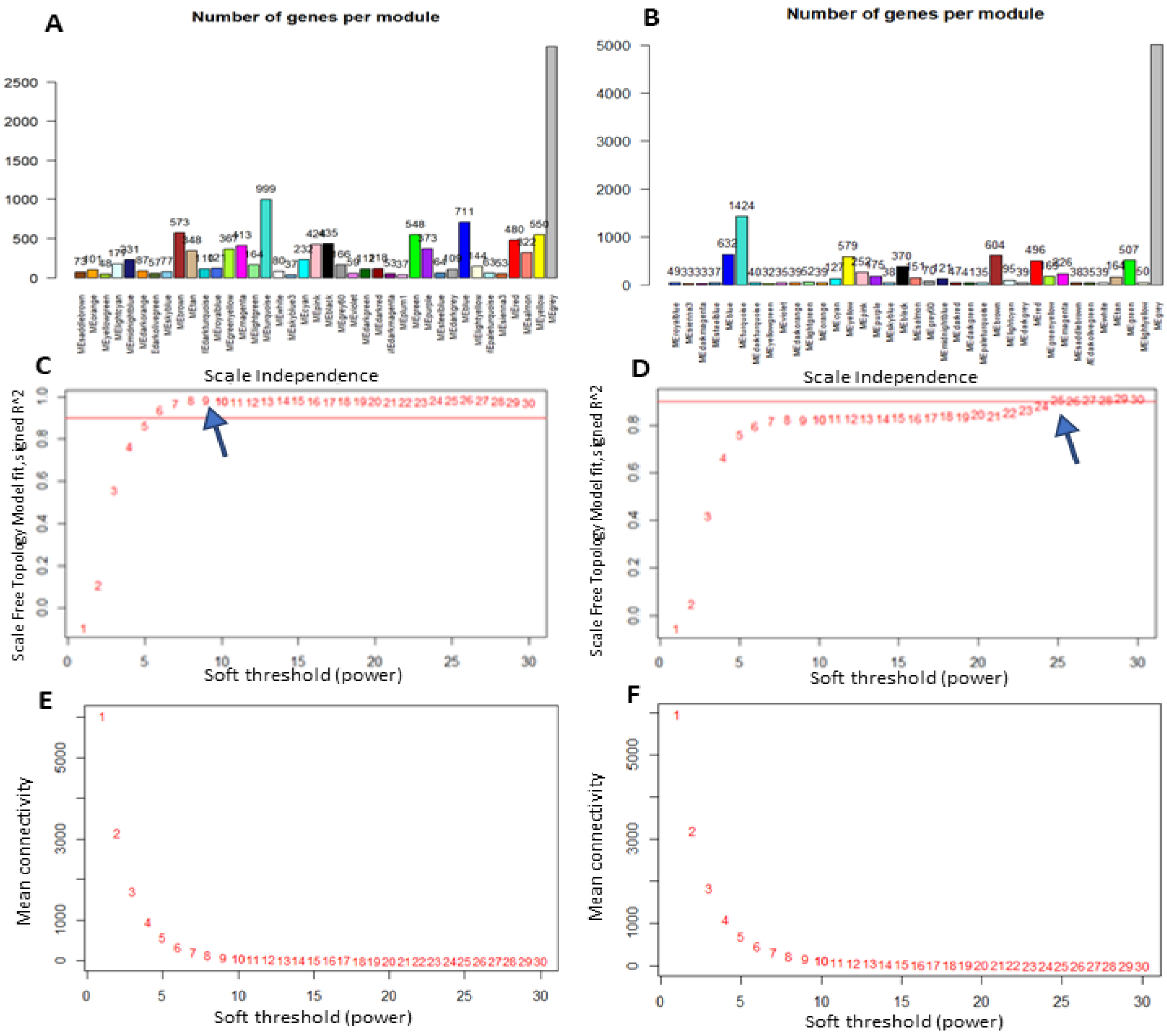

Fig. S2: *WGCNA. Diagnostic plots showing various beta fits to reach a scale-free topology network. Analysis of scale-free network topology using different soft-thresholding power on 4 days transcriptomics data. A and B show the numbers of genes in every module, which was given an arbitrary colour. C and D show the scale free fit index (y-axis) whereas E and F show the mean connectivity (degree, y-axis).*

Table S1 *The number of differentially expressed genes generated by DESeq2 in three different design group(linear, low-, and high- dose rate responsive group). Except for Ctrl vs 1 which is a*

low dose rate-responsive group, the data from 8 days has more DEGs than from 4 days in the high dose-rate responsive group (1 vs 100) and the linear model.

**Design group of DESeq2 (Types of DEGs)**		**Number of DEGs**	
		**4 days**	**8 days**
**Linear model**		312	1262
**Linear combination /Contrasts (mGy/h)**	**Low dose rate responsive (0 vs 1)**	233	49
	**High dose rate responsive (1 vs 100)**	623	1153

A

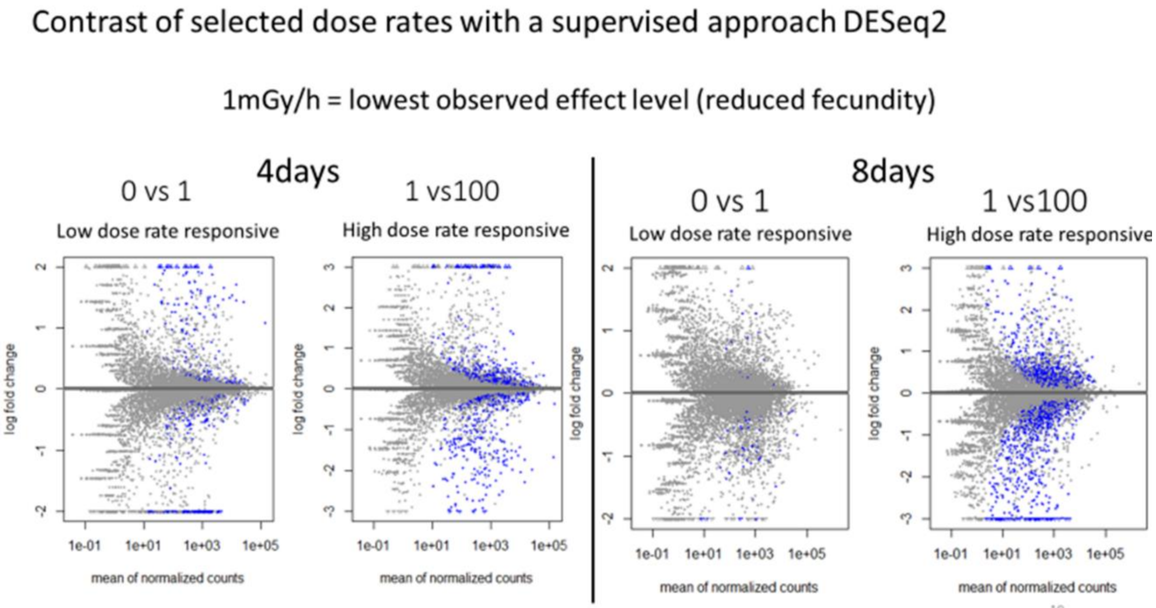

B

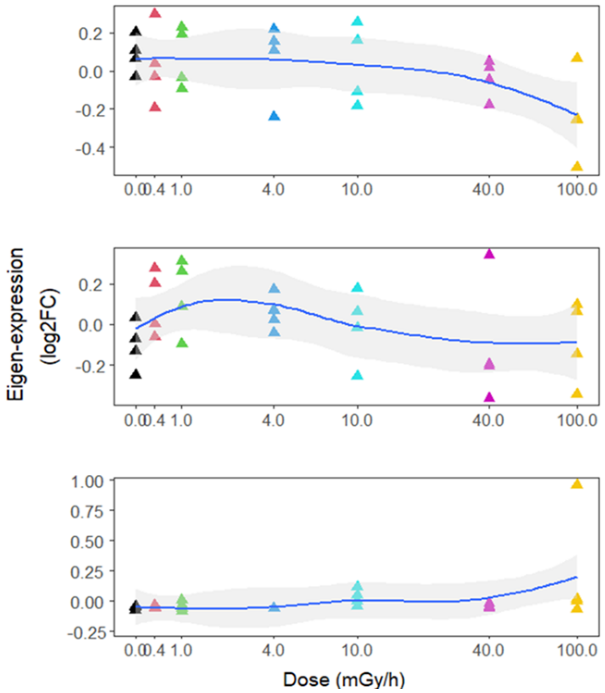

Fig. S3: *(A) Volcano plots showing DEGs from linear combinations (DESeq2). (B) Examples of the eigengene expression profiles showing monotonic (top), and non-monotonic: low-(middle) and high (bottom) dose rates responses. Eigengene module is the first principal component which best-represents the gene expression profile of a module.*

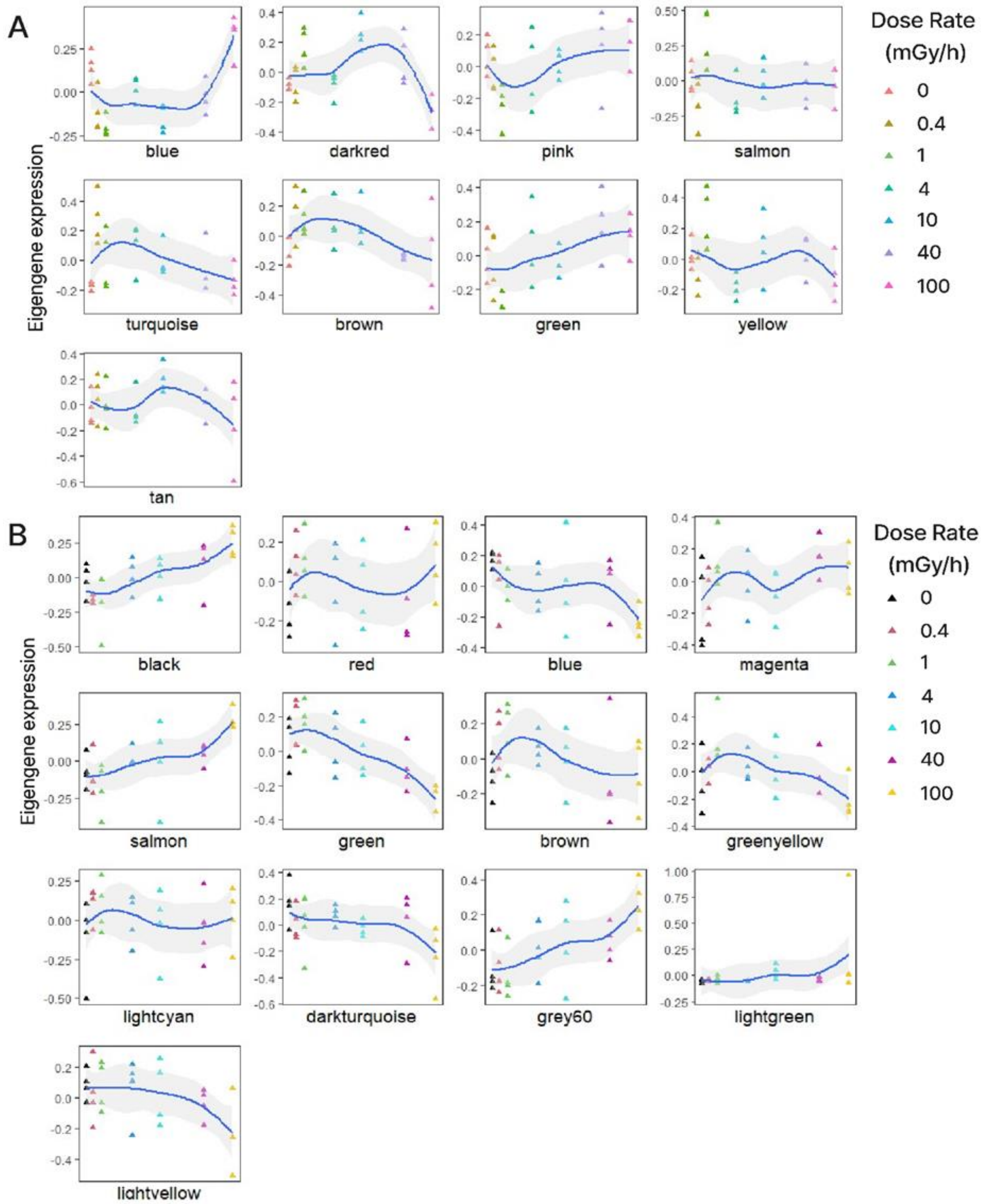

Fig. S4: *All significant modules in the 4-days (A) and 8-days (B) gamma radiation exposure data. Each module is represented by its eigengene (the first principal component of the module); y-axis showing the expression (Log2FC), x-axis showing the dose rates.*

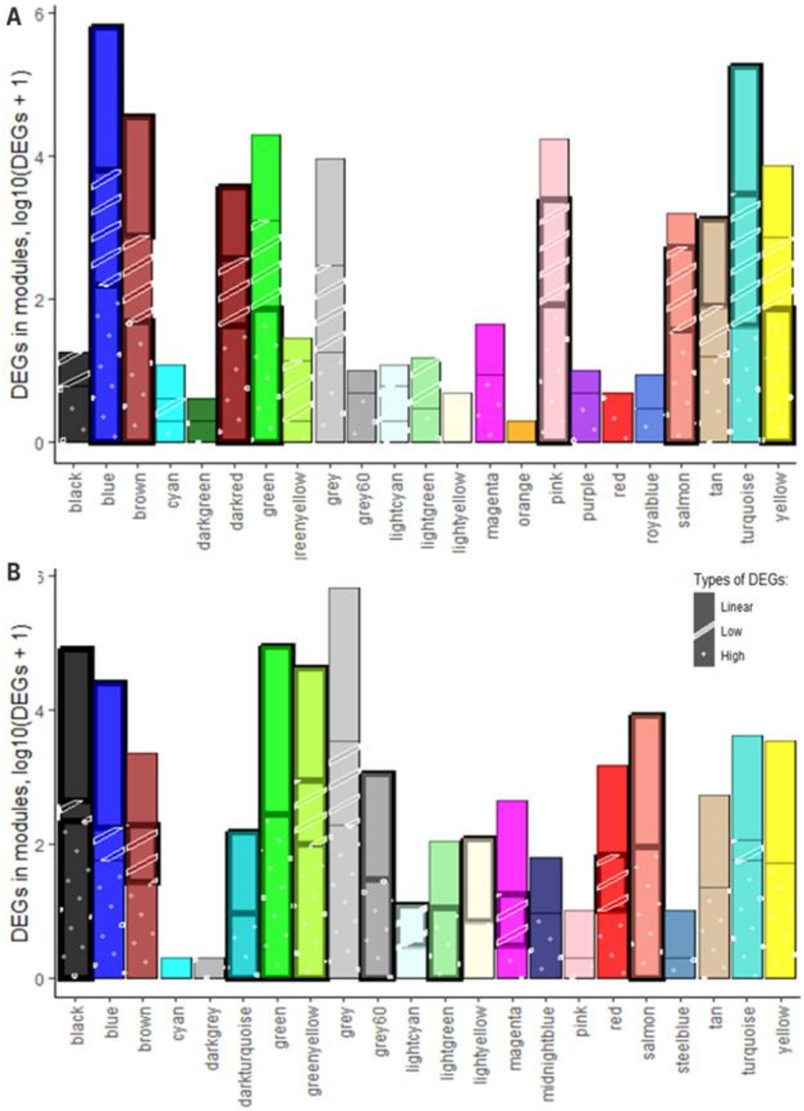

Fig. S5: *Significant modules detected through the overlapped of WGCNA and DESeq2 though the Fisher' s Exact test. The number of DEGs in modules from 4 days (A) and 8 days (B) of radiation exposure were showed by the y-axis and the name of modules were indicated by the x-axis. Three types of DEGs were identified: monotonic increase/decrease (termed "Linear" for simplicity), low dose responsive (Low) and high dose responsive (High). The thick black borders mark modules that are significantly enriched in DEGs.*

Table S2: *TF (transcription factors) orthologs and their corresponding modules from the data of 4 days of gamma radiation exposure. Gene symbols and gene names followed the nomenclatures of D. melanogaster as documented in Flybase. TFs (transcription factors) are considered activated if a corresponding enriched motif was found and the ortholog genes which encoded for the TFs were present in that module.*

**Transcript.ID**	**Gene.Symbol**	**Gene.name**	**Module.name**	**Activated?**
XM_032933675.1	*lola*	*longitudinals lacking*	blue	N
XM_032939826.1	*NK7.1*	*NK7.1*	blue	N
XM_032925696.1	*btd*	*buttonhead*	brown	N
XM_032937296.1	*CG32532*	uncharacterized protein	brown	N
XM_032937314.1	*ken*	*ken and barbie*	brown	Y
XM_032943003.1	*HHEX*	*Hematopoietically expressed homeobox*	brown	Y
XM_032929238.1	*Klf15*	*Kruppel-like factor 15*	pink	N
XM_032927636.1	*Blimp-1*	*Blimp-1*	pink	Y
XM_032935662.1	*ap*	*apterous*	pink	Y
XM_032923201.1	*br*	*broad*	pink	Y
XM_032928081.1	*pnr*	*pannier*	pink	Y
XM_032936003.1	*ken*	*ken and barbie*	salmon	Y
XM_032922483.1	*Pph13*	*PvuII-PstI homology 13*	salmon	N
XM_032929417.1	*exex*	*extra-extra*	salmon	Y
XM_032922964.1	*Awh*	*Arrowhead*	turquoise	Y
XM_032927749.1	*Awh*	*Arrowhead*	turquoise	Y
XM_032933804.1	*Awh*	*Arrowhead*	turquoise	Y

XM_032936275.1	*lola*	*longitudinals lacking*	turquoise	N
XM_032936557.1	*ken*	*ken and barbie*	turquoise	Y
XM_032930893.1	*Lim1*	*LIM homeobox 1*	turquoise	Y
XM_032940896.1	*ttk*	*tramtrack*	yellow	N

Table S3: *TF orthologs and their corresponding modules from the data of 8 days of gamma radiation exposure. Gene symbols and gene names followed the nomenclatures of D. melanogaster as documented in Flybase. TFs are considered activated if a corresponding enriched motif was found and the ortholog genes which encoded for the TFs were present in that module.*

**Transcript.ID**	**Gene.Symbol**	**Gene.name**	**Module.name**	**Activated?**
XM_032930933.1	*Sp1*	*Sp1*	black	N
XM_032924480.1	*br*	*broad*	black	Y
XM_032926776.1	*CG11617*	uncharacterized protein	black	N
XM_032929714.1	*ara*	*araucan*	black	N
XM_032926090.1	*Dbx*	*Dbx*	black	Y
XM_032929673.1	*lab*	*labial*	black	N
XM_032934768.1	*C15*	*C15*	black	N
XM_032937297.1	*opa*	*odd paired*	black	Y
XM_032938232.1	*E5*	*E5*	black	Y
XM_032941236.1	*Awh*	*Arrowhead*	black	N
XM_032936085.1	*ken*	*ken and barbie*	blue	N
XM_032934852.1	*ken*	*ken and barbie*	blue	N
XM_032927420.1	*ken*	*ken and barbie*	blue	N
XM_032943597.1	*CG7368*	uncharacterized protein	blue	Y

XM_032929033.1	*ttk*	*tramtrack*	brown	Y
XM_032935044.1	*ap*	*apterous*	brown	Y
XM_032923756.1	*lola*	*longitudinals lacking*	brown	Y
XM_032936275.1	*lola*	*longitudinals lacking*	brown	Y
XM_032929670.1	*lab*	*labial*	green	N
XM_032941531.1	*br*	*broad*	green	Y
XM_032934371.1	*ken*	*ken and barbie*	greenyellow	N
XM_032942013.1	*C15*	*C15*	grey60	N
XM_032934613.1	*fru*	*fruitless*	magenta	Y
XM_032925433.1	*br*	*broad*	red	Y
XM_032941351.1	*Lmx1a*	*LIM homeobox transcription factor 1 alpha*	red	Y
XM_032922964.1	*Lmx1a*	*LIM homeobox transcription factor 1 alpha*	red	Y
XM_032930893.1	*Lim1*	*LIM homeobox 1*	red	N
XM_032926422.1	*Sp1*	*Sp1*	red	N
XM_032920460.1	*ken*	*ken and barbie*	red	N
XM_032921220.1	*repo*	*reversed polarity*	red	N
XM_032928317.1	*exd*	*extradenticle*	salmon	Y
XM_032922650.1	*ap*	*apterous*	salmon	Y
XM_032923766.1	*CG11294*	uncharacterized protein	salmon	N
XM_032921442.1	*lab*	*labial*	salmon	N
XM_032925696.1	*btd*	*buttonhead*	salmon	Y

XM_032936557.1	*ken*	*ken and barbie*	salmon	N
XM_032921036.1	*Vsx2*	*Visual system homeobox 2*	salmon	N
XM_032938902.1	*Lim1*	*LIM homeobox 1*	salmon	N
XM_032923740.1	*pnr*	*pannier*	salmon	N

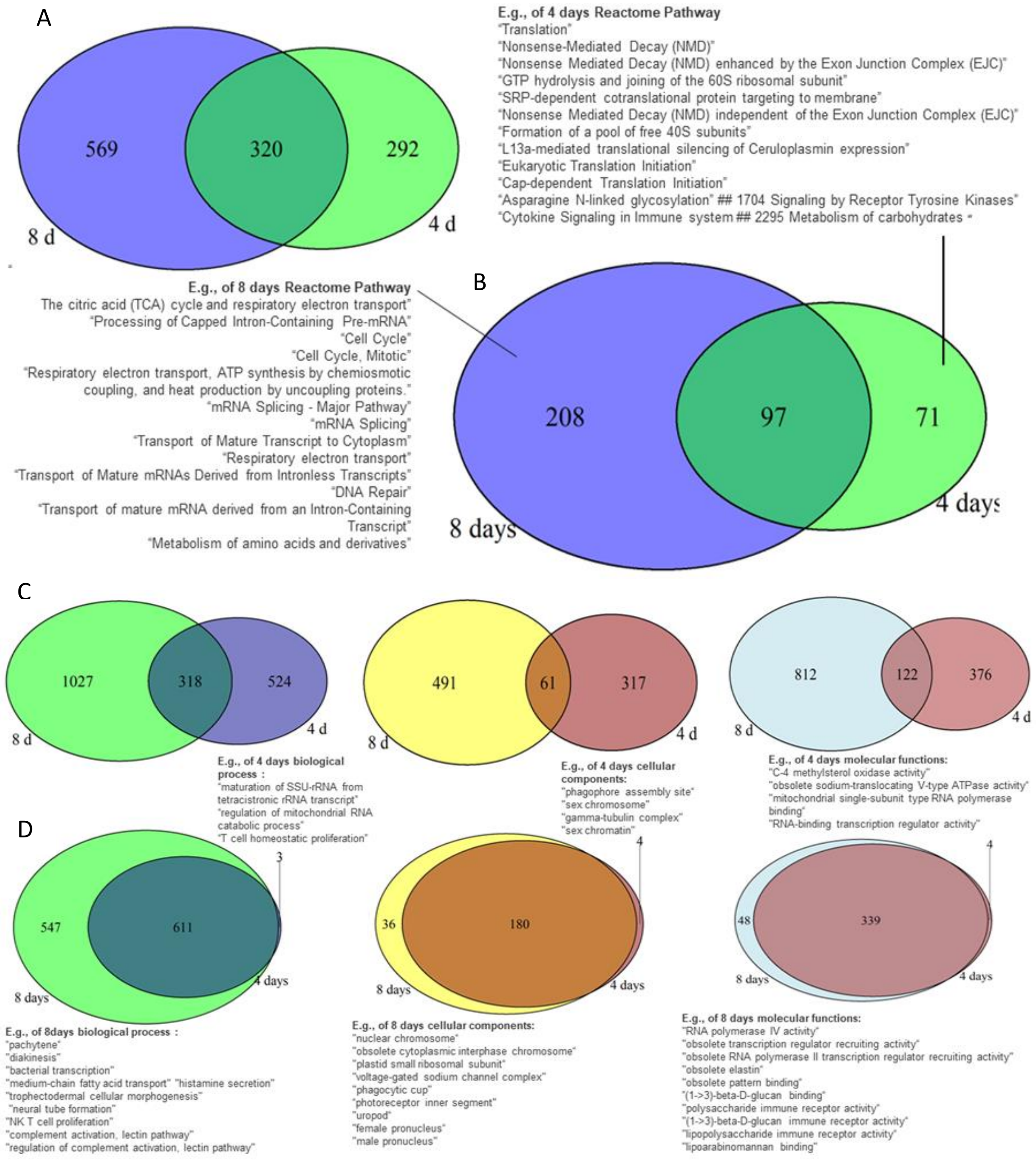

Fig. S6: *Venn diagram shows number of significantly enriched GO terms for 4 days vs 8 days radiation exposure (A-B) and the enriched Reactome pathway (p-value < 0.05) (C-D). The six Venn diagrams above depict the number of genes that were involved in the Reactome overrepresented pathway analysis (A) and the number of pathway dedicated to each exposure period (B). The top enriched Reactome pathway are labelled by different exposure periods. The six Venn diagrams below are the output from Reactome PA, the number of genes involved in GO analysis (C) and the labels indicate the top enriched pathways from each period (D).*

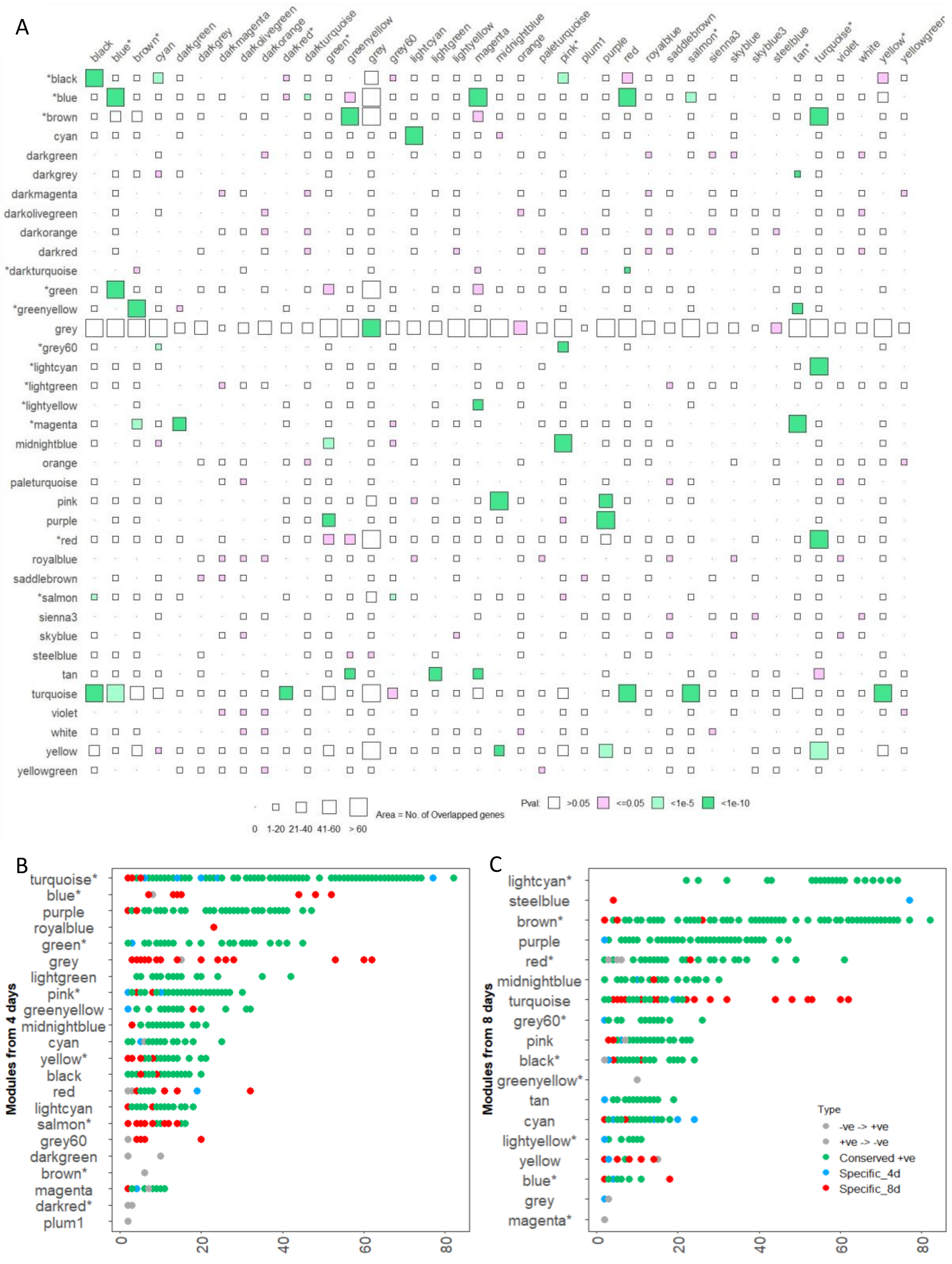

Fig. S7*: (A) Statistically significant overlap in gene content between modules discovered in the 4 days and 8 days data. Modules with significant changes in expression are marked with an asterisk (*). (B-C) The centrality (number of connections) of genes in the DiCE network grouped by module: 4 days (left) and 8 days (right). Genes can be: positively correlated in both exposure*

periods (Conserved +ve), positively correlated in one period and negative in the other (Differentiated) or correlated only in 4 days exposure (specific_4d) or only in 8 days (specific_8d).

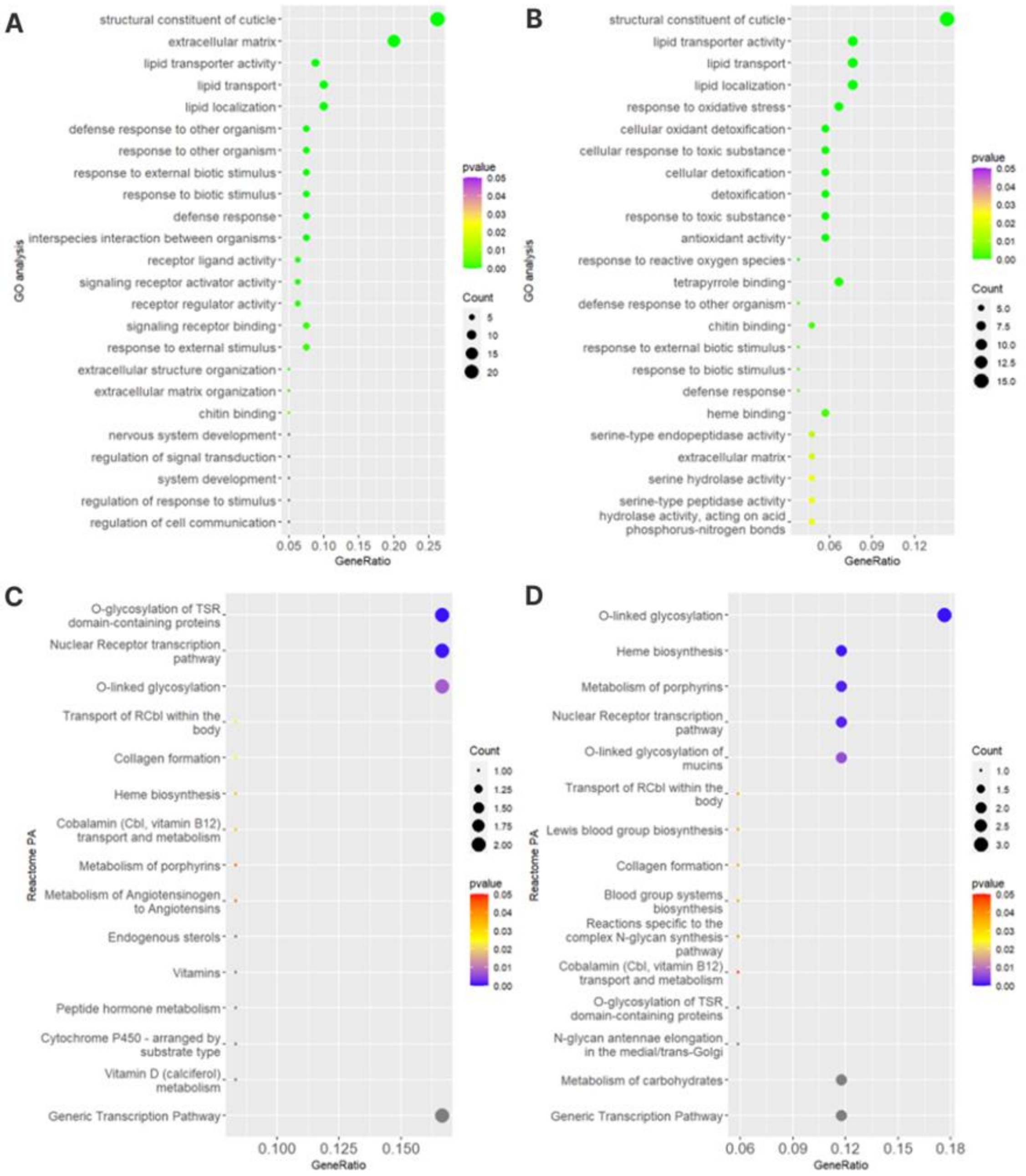

Fig. S8: *Functional annotation of DICE selected genes from 4 and 8 days with GO enrichment analysis (top A and B) and Reactome PA (bottom C and D).*

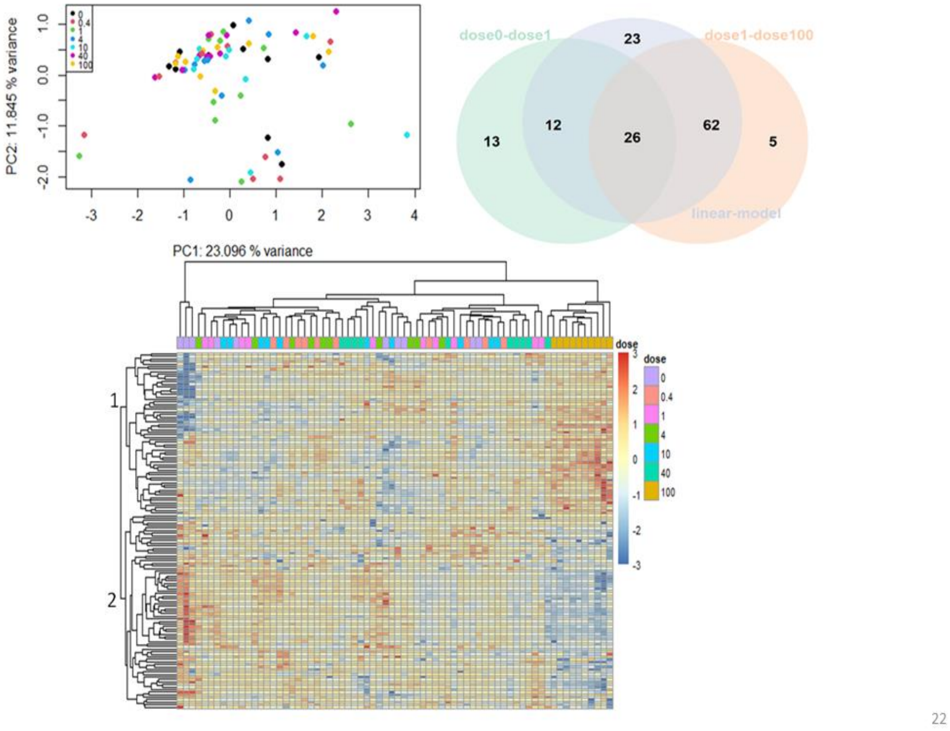

Fig. S9: *Plots show the initial analysis on metabolites abundance. (A) PCA (Principal Component Analysis) plot showed the sample of metabolites in 2D plane spanned by the first two principal components which explained the most variance. No clustering pattern observed indicates an extremely small difference between samples. (B) Venn diagram comparing the numbers of DEMs shared and uniquely existing between low dose-responsive, high-dose responsive and linear model groups. (C) Heatmap showing the gene expression of all metabolites and all samples; red to blue colour scale represents high to low gene expression and the colour of dose rates was represented by the legend on the right.*

Fig. S10: Paintomics integrated pathway enrichment analysis

A

Low dose rate responsive group (8 days)

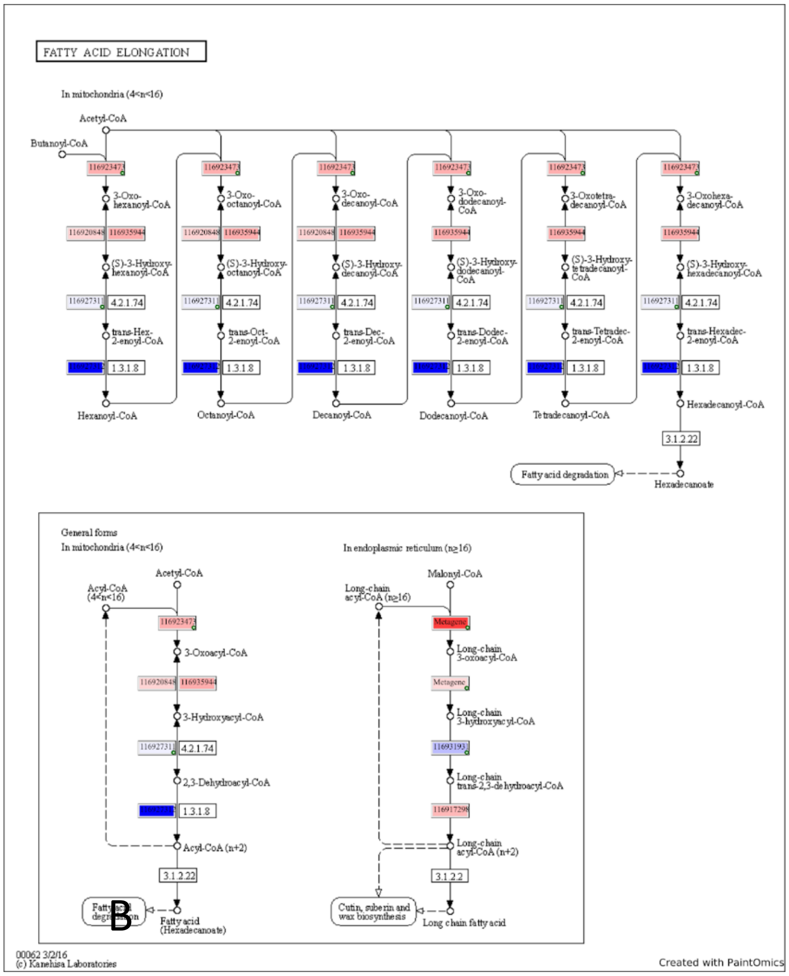

High dose rate responsive group (8 days)

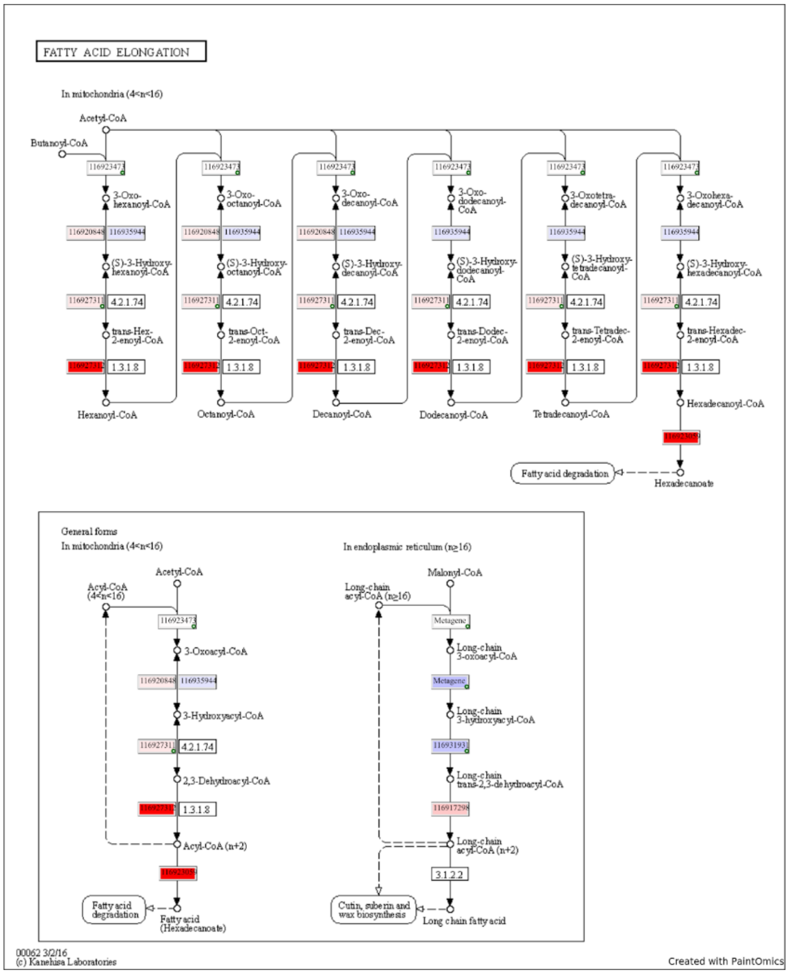

B

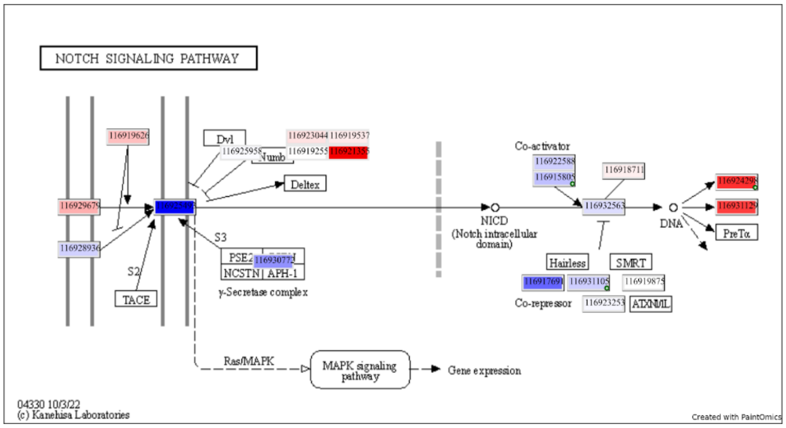

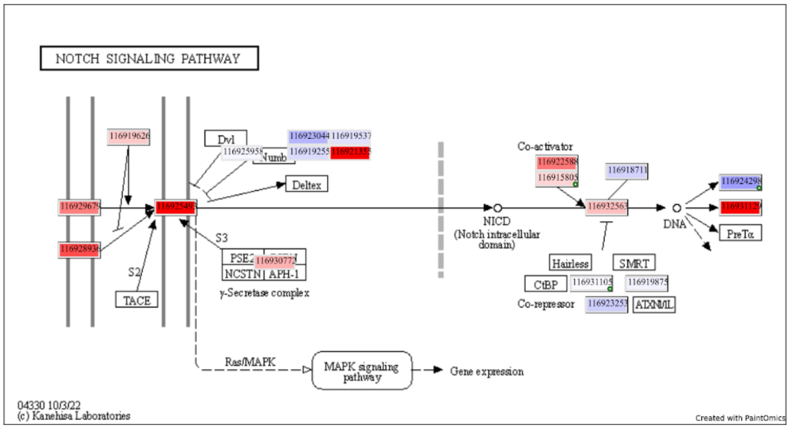

C

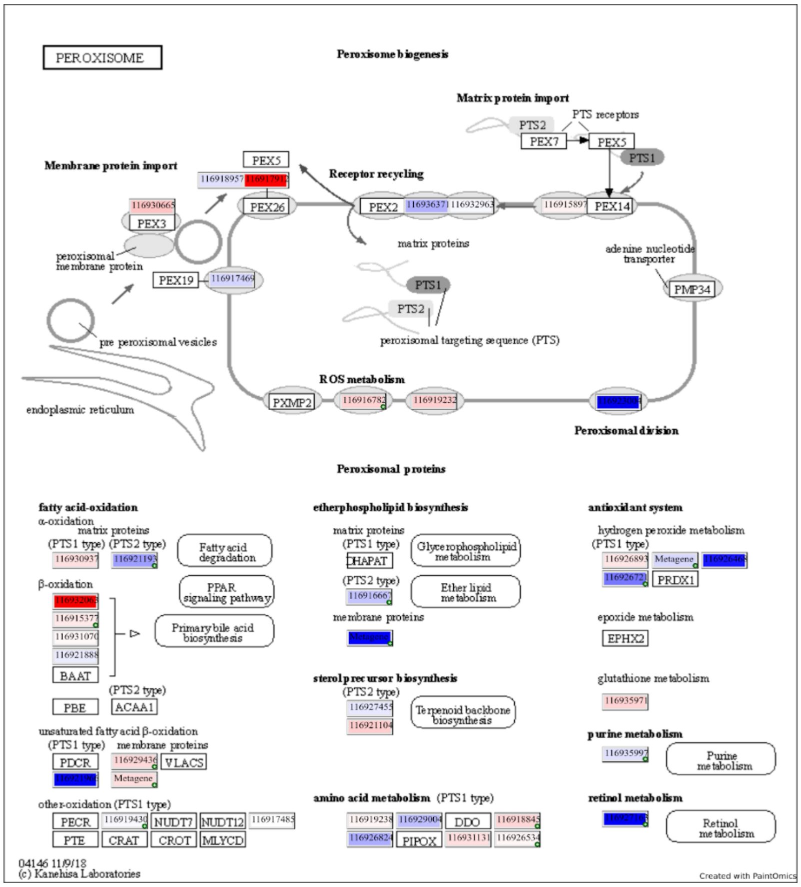
PEROXISOME
Peroxisome biogenesis
Matrix protein import
PTS receptors
PTS2
PEX7
PEX5
PTS1
Membrane protein import
Receptor recycling
PEX5
116918957
PEX26
PEX2
116936371
116932963
116915897
PEX14
116930665
PEX3
matrix proteins
adenine nucleotide transporter
peroxisomal membrane protein
PEX19
116917469
PMP34
PTS1
PTS2
pre peroxisomal vesicles
peroxisomal targeting sequence (PTS)
ROS metabolism
endoplasmic reticulum
PXMP2
116916782
116919232
Peroxisomal division
Peroxisomal proteins
fatty acid-oxidation
α-oxidation
matrix proteins
(PTS1 type) (PTS2 type)
116930937
Fatty acid degradation
β-oxidation
PPAR signaling pathway
116915377
116931070
116921888
Primary bile acid biosynthesis
BAAT
(PTS2 type)
PBE
ACAA1
unsaturated fatty acid β-oxidation
(PTS1 type) membrane proteins
PDCR
116929436
VLACS
Metagene
other-oxidation (PTS1 type)
PECR
116919436
NUDT7
NUDT12
116917485
PTE
CRAT
CROT
MLYCD
etherphospholipid biosynthesis
matrix proteins
(PTS1 type)
DHAPAT
Glycerophospholipid metabolism
(PTS2 type)
116916667
Ether lipid metabolism
membrane proteins
sterol precursor biosynthesis
(PTS2 type)
116927455
116921104
Terpenoid backbone biosynthesis
amino acid metabolism (PTS1 type)
116919238
116929004
DDO
116918845
116926824
PIPOX
116931131
116926534
antioxidant system
hydrogen peroxide metabolism
(PTS1 type)
116926893
Metagene
116926722
PRDX1
epoxide metabolism
EPHX2
glutathione metabolism
116935971
purine metabolism
116935997
Purine metabolism
retinol metabolism
Retinol metabolism
04146 11/9/18
(c) Kanehisa Laboratories
Created with PaintOmics

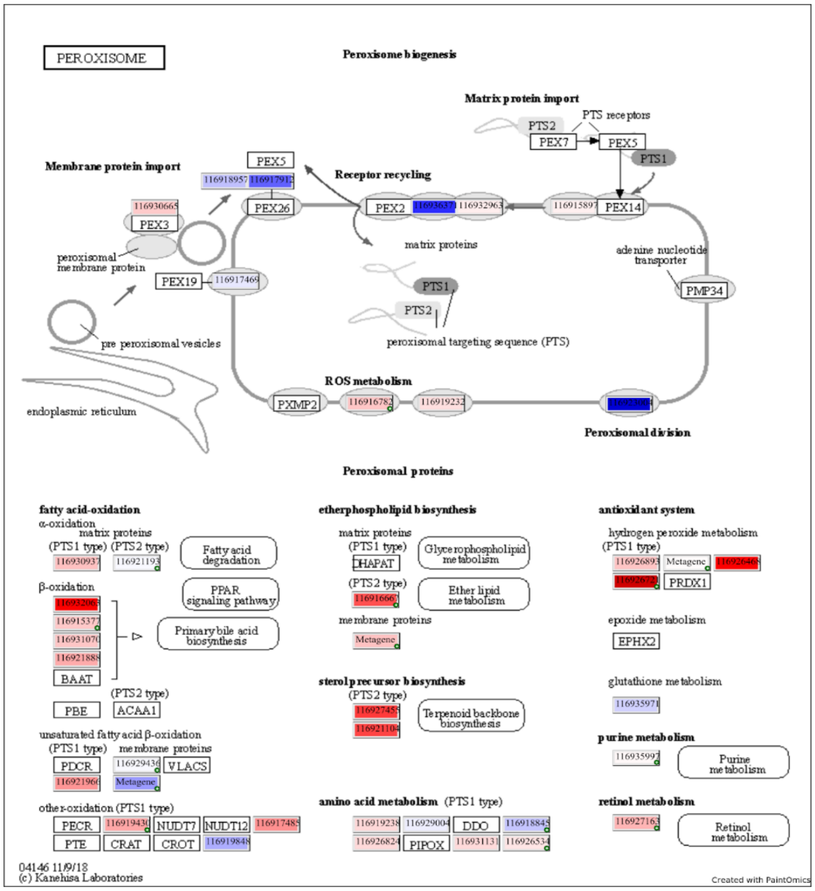
PEROXISOME
Peroxisome biogenesis
Matrix protein import
PTS receptors
PTS2
PEX7
PEX5
PTS1
Membrane protein import
Receptor recycling
PEX5
116918957
PEX26
PEX2
116932963
116915897
PEX14
116930665
PEX3
matrix proteins
adenine nucleotide transporter
peroxisomal membrane protein
PEX19
116917469
PMP34
pre peroxisomal vesicles
peroxisomal targeting sequence (PTS)
ROS metabolism
endoplasmic reticulum
PXMP2
116916782
116919232
Peroxisomal division
Peroxisomal proteins
fatty acid-oxidation
α-oxidation
matrix proteins
(PTS1 type) (PTS2 type)
116930937
116921193
Fatty acid degradation
β-oxidation
PPAR signaling pathway
116915377
116931070
116921888
Primary bile acid biosynthesis
BAAT
(PTS2 type)
PBE
ACAA1
unsaturated fatty acid β-oxidation
(PTS1 type) membrane proteins
PDCR
116929436
VLACS
116921966
Metagene
other-oxidation (PTS1 type)
PECR
116919436
NUDT7
NUDT12
116917485
PTE
CRAT
CROT
116919848
etherphospholipid biosynthesis
matrix proteins
(PTS1 type)
DHAPAT
Glycerophospholipid metabolism
(PTS2 type)
116916667
Ether lipid metabolism
membrane proteins
Metagene
sterol precursor biosynthesis
(PTS2 type)
116927455
116921104
Terpenoid backbone biosynthesis
amino acid metabolism (PTS1 type)
116919238
116929004
DDO
116918845
116926824
PIPOX
116931131
116926534
antioxidant system
hydrogen peroxide metabolism
(PTS1 type)
116926893
Metagene
PRDX1
epoxide metabolism
EPHX2
glutathione metabolism
116935971
purine metabolism
116935997
Purine metabolism
retinol metabolism
116927163
Retinol metabolism
04146 11/9/18
(c) Kanehisa Laboratories
Created with PaintOmics

D

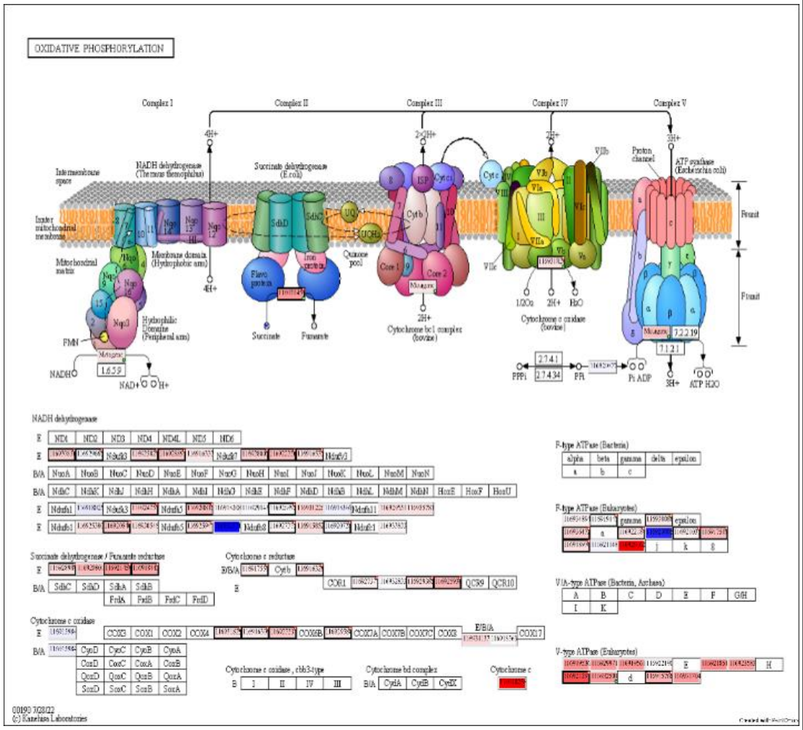
OXIDATIVE PHOSPHORYLATION
Complex I
Complex II
Complex III
Complex IV
Complex V
NADH dehydrogenase
Succinate dehydrogenase
Cytochrome c oxidase
F-type ATPase (Bacteria)
F-type ATPase (Eukaryotes)
V/A-type ATPase (Bacteria, Archaea)
V-type ATPase (Eukaryotes)
Cytochrome c reductase
Cytochrome c oxidase, cbb3-type
Cytochrome bd complex
(c) Kanehisa Laboratories

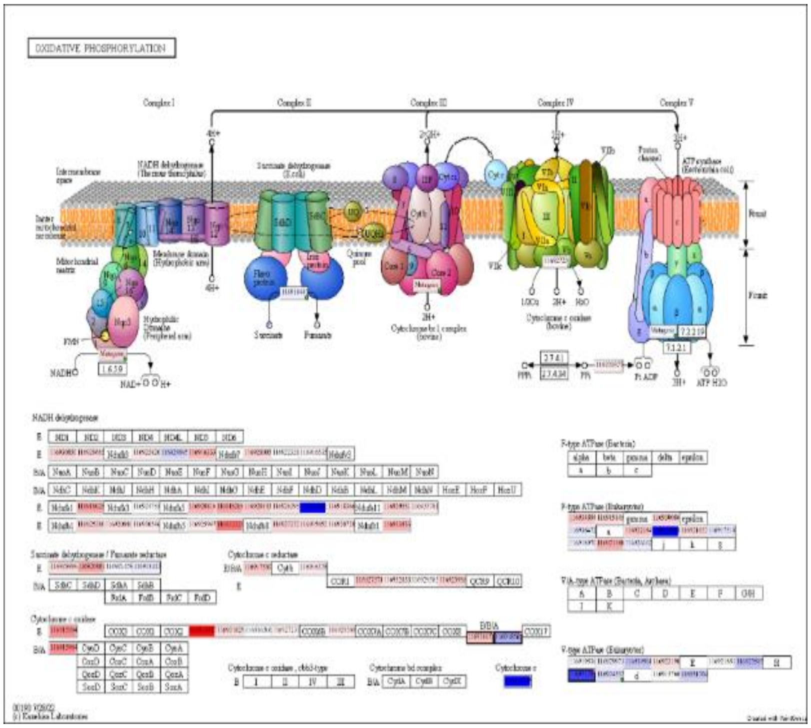
OXIDATIVE PHOSPHORYLATION
(c) Kanehisa Laboratories

E

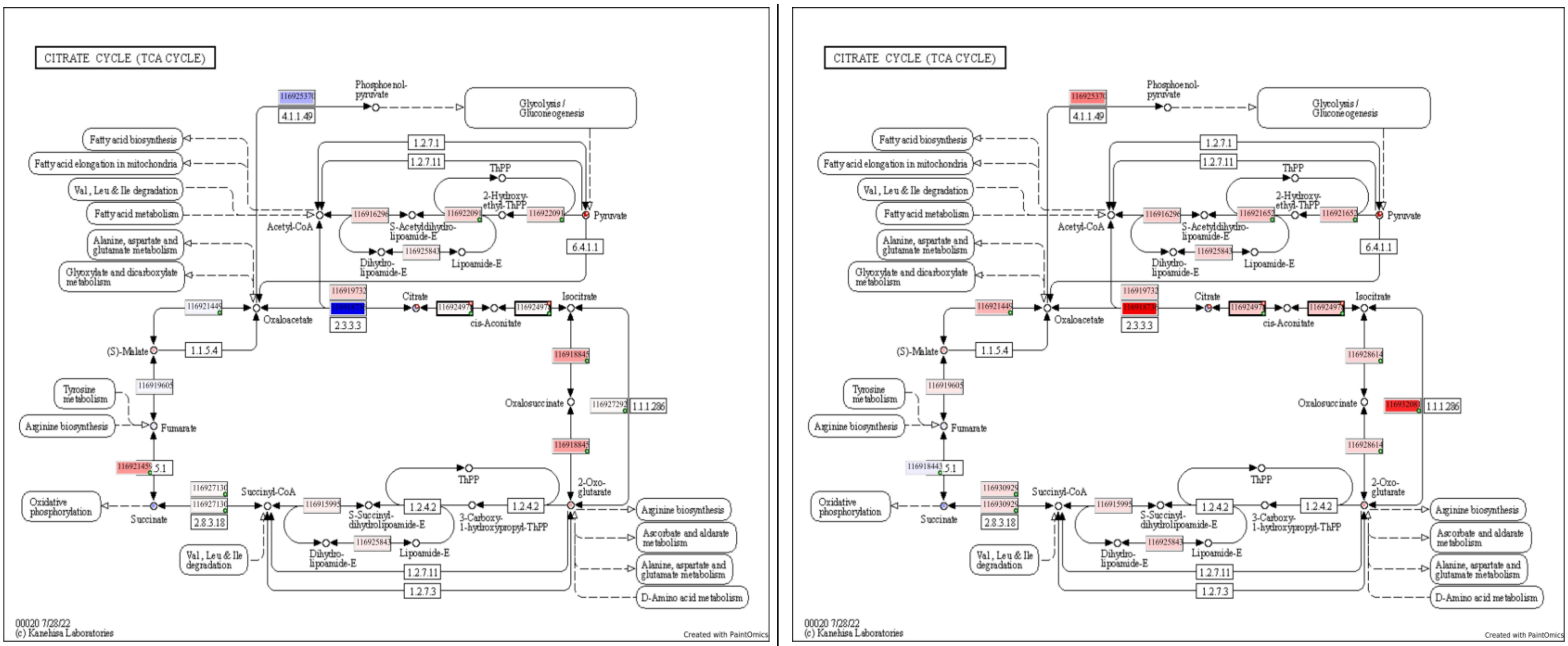
CITRATE CYCLE (TCA CYCLE)
Phosphoenol-pyruvate
Glycolysis / Gluconeogenesis
Fatty acid biosynthesis
Fatty acid elongation in mitochondria
Val, Leu & Ile degradation
Fatty acid metabolism
Alanine, aspartate and glutamate metabolism
Glyoxylate and dicarboxylate metabolism
4.1.1.49
1.2.7.1
1.2.7.11
ThPP
2-Hydroxy-ethyl-ThPP
Pyruvate
Acetyl-CoA
S-Acetyldihydro-lipoamide-E
Dihydro-lipoamide-E
Lipoamide-E
6.4.1.1
Citrate
Isocitrate
cis-Aconitate
Oxaloacetate
2.3.3.3
(S)-Malate
1.1.5.4
Tyrosine metabolism
Arginine biosynthesis
Fumarate
Oxalosuccinate
1.1.1.286
Oxidative phosphorylation
Succinate
Succinyl-CoA
2.8.3.18
S-Succinyl-dihydrolipoamide-E
3-Carboxy-1-hydroxypropyl-ThPP
1.2.4.2
2-Oxo-glutarate
Ascorbate and aldarate metabolism
D-Amino acid metabolism
1.2.7.3
00020 7/28/22
(c) Kanehisa Laboratories
Created with PaintOmics

F

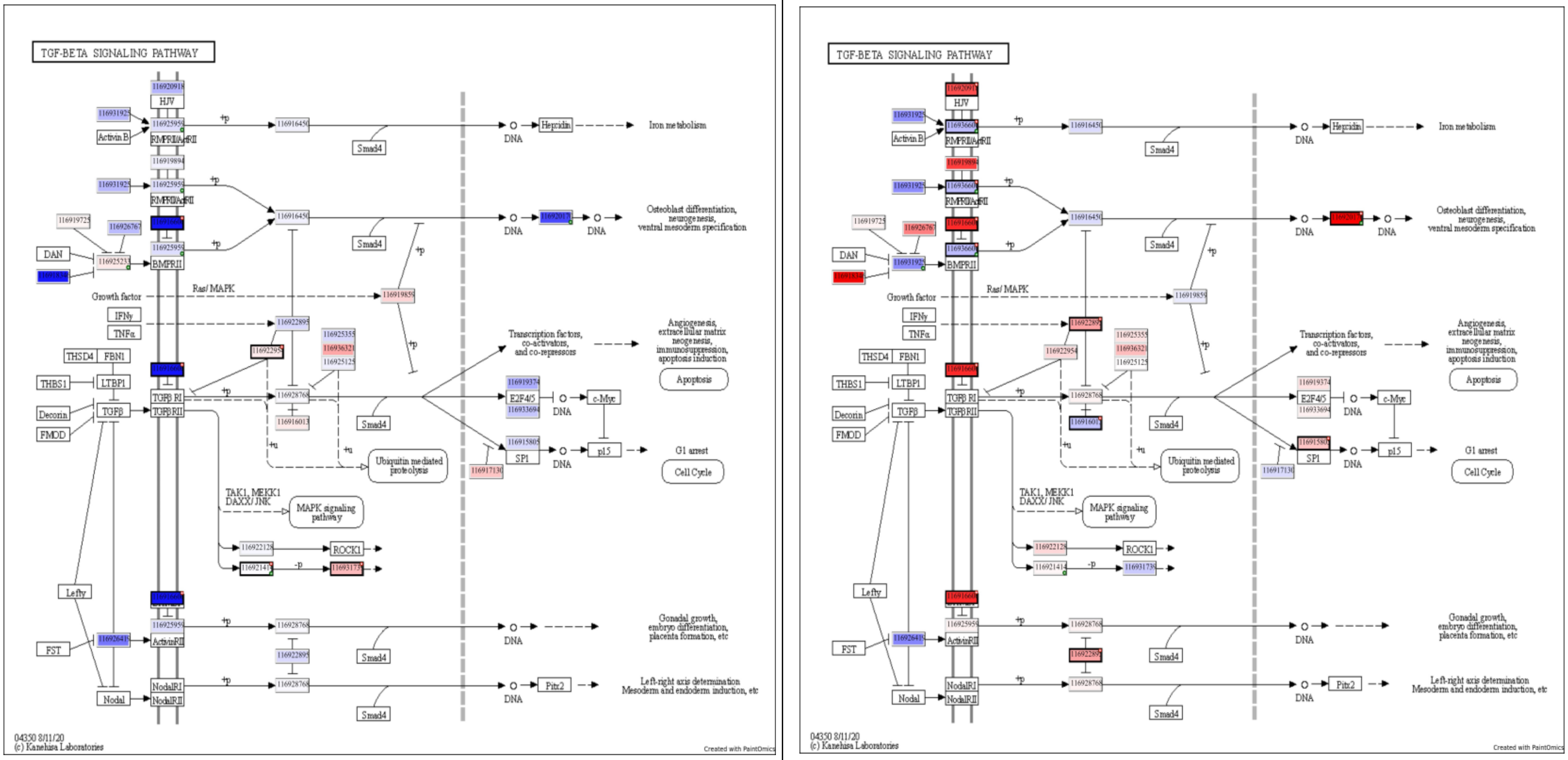
TGF-BETA SIGNALING PATHWAY
HJV
Activin B
RMPRII/ActRII
Hepcidin
Iron metabolism
Smad4
DNA
Osteoblast differentiation, neurogenesis, ventral mesoderm specification
DAN
BMPRII
Growth factor
Ras/ MAPK
IFNγ
TNFα
THSD4
FBN1
THBS1
LTBP1
Decorin
TGFβ
FMOD
TGFβ RI
TGFβ RII
Transcription factors, co-activators, and co-repressors
Angiogenesis, extracellular matrix neogenesis, immunosuppression, apoptosis induction
Apoptosis
E2F4/5
c-Myc
SP1
p15
G1 arrest
Cell Cycle
Ubiquitin mediated proteolysis
TAK1, MEKK1 DAXX/JNK
MAPK signaling pathway
ROCK1
Lefty
FST
ActivinRII
Gonadal growth, embryo differentiation, placenta formation, etc
Nodal
NodalRI
NodalRII
Pitx2
Left-right axis determination Mesoderm and endoderm induction, etc
04350 8/11/20
(c) Kanehisa Laboratories
Created with PaintOmics

G

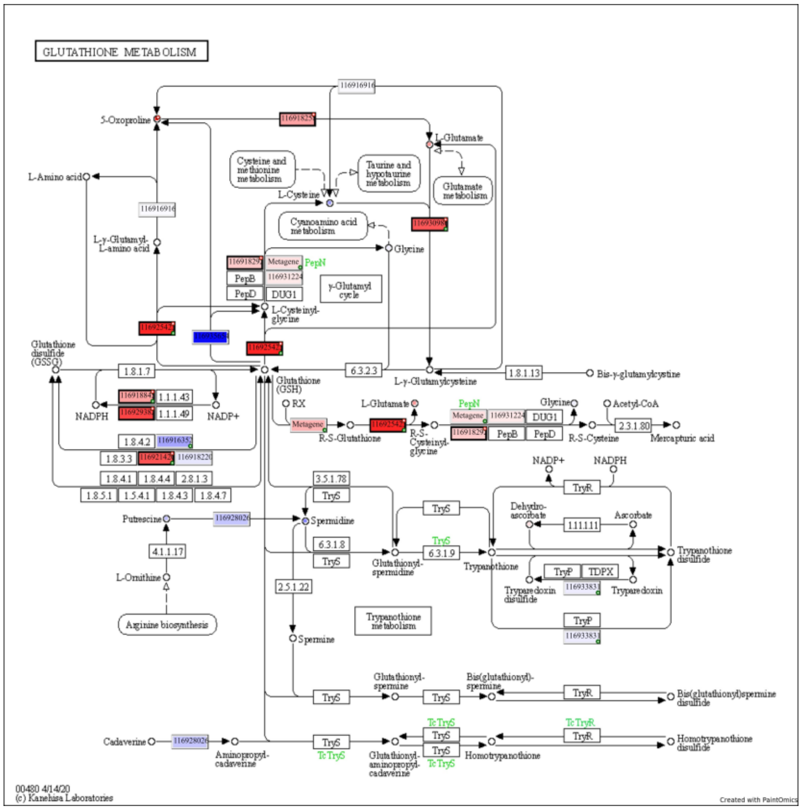
GLUTATHIONE METABOLISM
5-Oxoproline
L-Glutamate
L-Amino acid
Cysteine and methionine metabolism
Taurine and hypotaurine metabolism
Glutamate metabolism
L-Cysteine
Cyanoamino acid metabolism
Glycine
L-γ-Glutamyl-L-amino acid
γ-Glutamyl cycle
L-Cysteinyl-glycine
Glutathione disulfide (GSSG)
Glutathione (GSH)
L-γ-Glutamylcysteine
Bis-γ-glutamylcystine
NADPH
NADP+
RX
R-S-Glutathione
R-S-Cysteinyl-glycine
R-S-Cysteine
Acetyl-CoA
Mercapturic acid
Putrescine
Spermidine
L-Ornithine
Arginine biosynthesis
Dehydro-ascorbate
Ascorbate
Glutathionyl-spermidine
Trypanothione
Trypanothione disulfide
Tryparedoxin disulfide
Tryparedoxin
Trypanothione metabolism
Spermine
Glutathionyl-spermine
Bis(glutathionyl)-spermine
Bis(glutathionyl)spermine disulfide
Cadaverine
Aminopropyl-cadaverine
Glutathionyl-aminopropyl-cadaverine
Homotrypanothione
Homotrypanothione disulfide
00480 4/14/20
(c) Kanehisa Laboratories
Created with PaintOmics

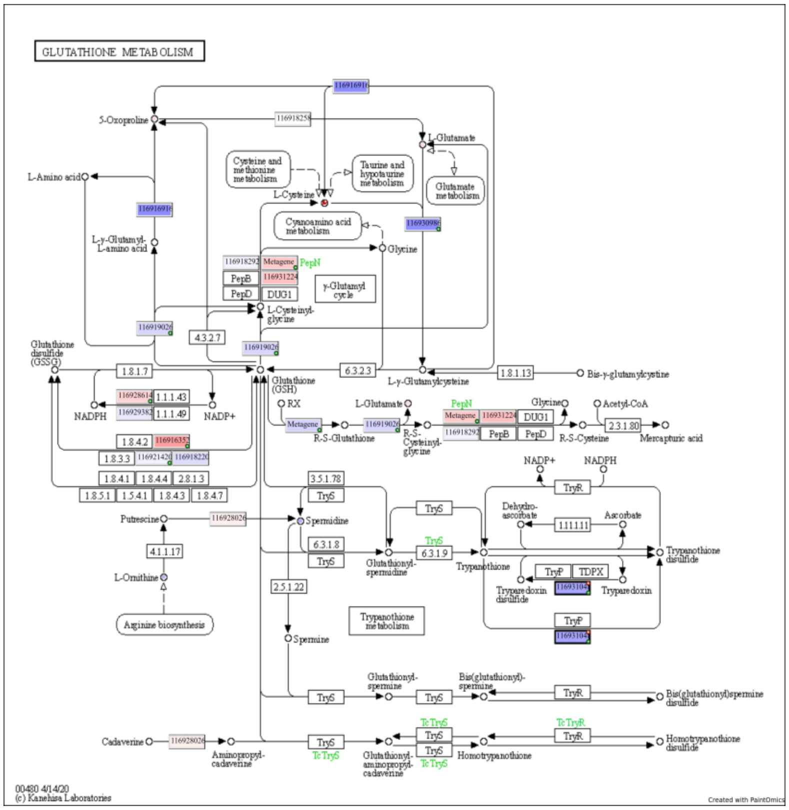
GLUTATHIONE METABOLISM
5-Oxoproline
L-Glutamate
L-Amino acid
Cysteine and methionine metabolism
Taurine and hypotaurine metabolism
Glutamate metabolism
L-Cysteine
Cyanoamino acid metabolism
Glycine
L-γ-Glutamyl-L-amino acid
γ-Glutamyl cycle
L-Cysteinyl-glycine
Glutathione disulfide (GSSG)
Glutathione (GSH)
L-γ-Glutamylcysteine
Bis-γ-glutamylcystine
NADPH
NADP+
RX
R-S-Glutathione
R-S-Cysteinyl-glycine
R-S-Cysteine
Acetyl-CoA
Mercapturic acid
Putrescine
Spermidine
L-Ornithine
Arginine biosynthesis
Dehydro-ascorbate
Ascorbate
Glutathionyl-spermidine
Trypanothione
Trypanothione disulfide
Tryparedoxin disulfide
Tryparedoxin
Trypanothione metabolism
Spermine
Glutathionyl-spermine
Bis(glutathionyl)-spermine
Bis(glutathionyl)spermine disulfide
Cadaverine
Aminopropyl-cadaverine
Glutathionyl-aminopropyl-cadaverine
Homotrypanothione
Homotrypanothione disulfide
00480 4/14/20
(c) Kanehisa Laboratories
Created with PaintOmics

H

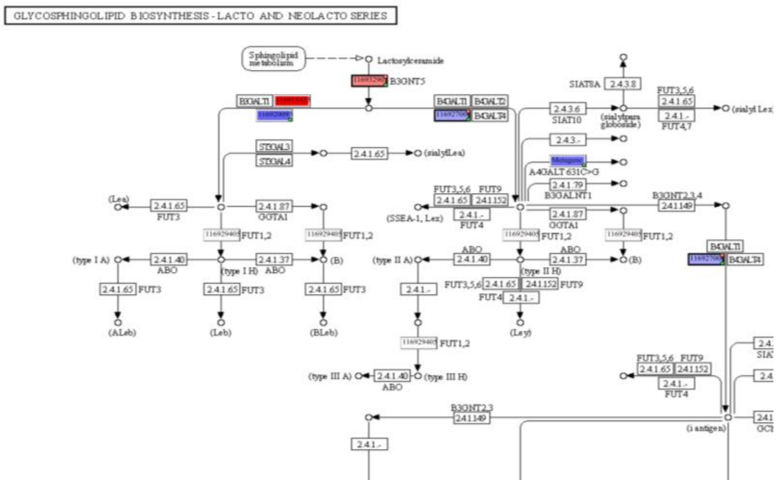
GLYCOSPHINGOLIPID BIOSYNTHESIS - LACTO AND NEOLACTO SERIES
Sphingolipid metabolism
Lactosylceramide
B3GNT5
(sialyl Lex)
(sialyl Lea)
(Lea)
(SSEA-1, Lex)
(type I A)
(type I H)
(B)
(type II A)
(type II H)
(ALeb)
(Leb)
(BLeb)
(Ley)
(type III A)
(type III H)
(i antigen)

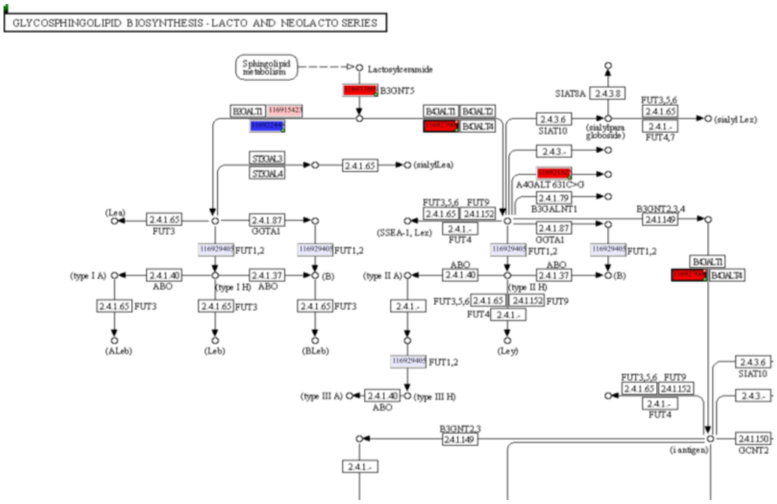
GLYCOSPHINGOLIPID BIOSYNTHESIS - LACTO AND NEOLACTO SERIES
Sphingolipid metabolism
Lactosylceramide
B3GNT5
(sialyl Lex)
(sialyl Lea)
(Lea)
(SSEA-1, Lex)
(type I A)
(type I H)
(B)
(type II A)
(type II H)
(ALeb)
(Leb)
(BLeb)
(Ley)
(type III A)
(type III H)
(i antigen)

|

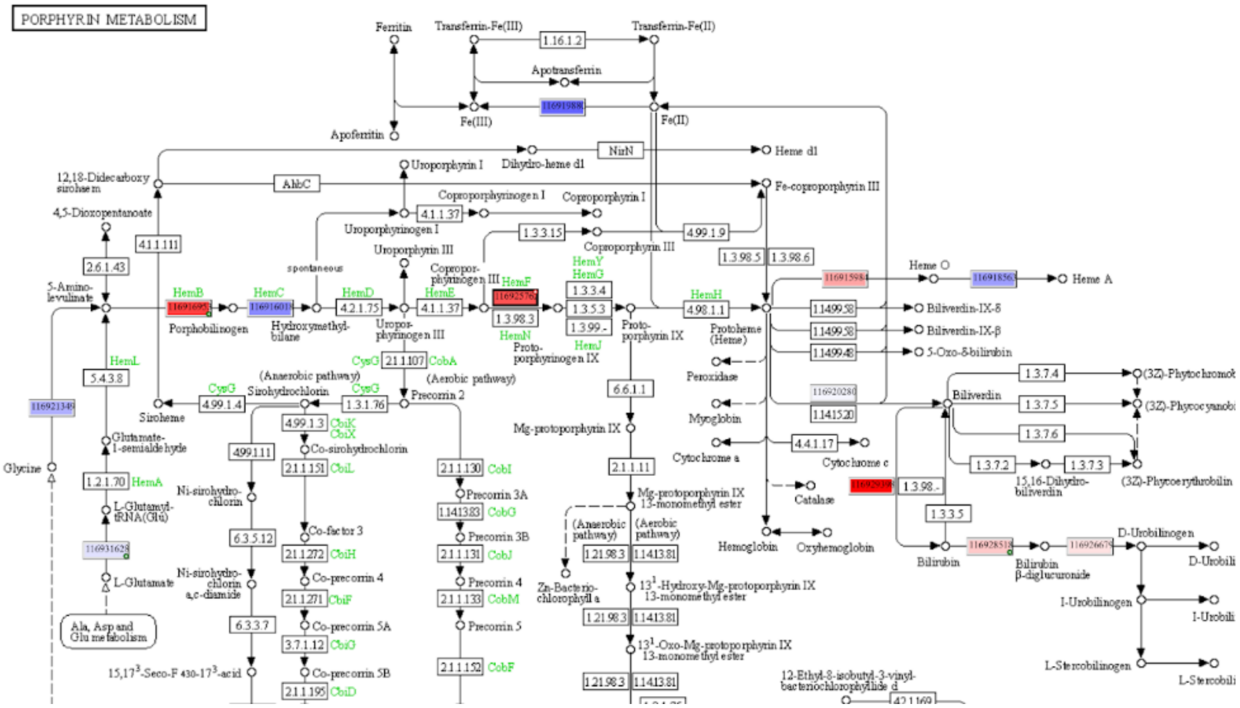
PORPHYRIN METABOLISM
Ferritin
Transferrin-Fe(III)
Transferrin-Fe(II)
1.16.1.2
Apotransferrin
Fe(III)
Fe(II)
Apoferritin
NirN
Dihydro-heme d1
Heme d1
Uroporphyrin I
12,18-Didecarboxy siroheam
AhbC
Fe-coproporphyrin III
4,5-Dioxopentanoate
Coproporphyrinogen I
Coproporphyrin I
4.1.1.37
Uroporphyrinogen I
1.3.3.15
4.99.1.9
Coproporphyrin III
Uroporphyrin III
spontaneous
5-Amino-levulinate
HemB
HemC
HemD
HemE
HemF
HemY
HemG
HemH
HemN
HemJ
HemL
HemA
Porphobilinogen
Hydroxymethyl-bilane
Uropor-phyrinogen III
Copropor-phyrinogen III
Proto-porphyrinogen IX
Proto-porphyrin IX
Protoheme (Heme)
Heme O
Heme A
Biliverdin-IX-δ
Biliverdin-IX-β
5-Oxo-δ-bilirubin
Peroxidase
Myoglobin
Cytochrome a
Cytochrome c
Catalase
Hemoglobin
Oxyhemoglobin
Biliverdin
Bilirubin
Bilirubin β-diglucuronide
D-Urobilinogen
I-Urobilinogen
L-Stercobilinogen
15,16-Dihydro-biliverdin
(3Z)-Phycoerythrobilin
Siroheme
Sirohydrochlorin
Precorrin 2
(Anaerobic pathway)
(Aerobic pathway)
Glycine
Glutamate-1-semialdehyde
L-Glutamyl-tRNA(Glu)
L-Glutamate
Ala, Asp and Glu metabolism
Mg-protoporphyrin IX
Mg-protoporphyrin IX 13-monomethyl ester
Zn-Bacterio-chlorophyll a
Co-sirohydrochlorin
Co-factor 3
Co-precorrin 4
Co-precorrin 5A
Co-precorrin 5B
Precorrin 3A
Precorrin 3B
Precorrin 4
Precorrin 5
Ni-sirohydro-chlorin
CysG
CobA
CbiK
CbiX
CbiL
CbiH
CbiF
CbiG
CbiD
CobI
CobG
CobJ
CobM
CobF

4 days

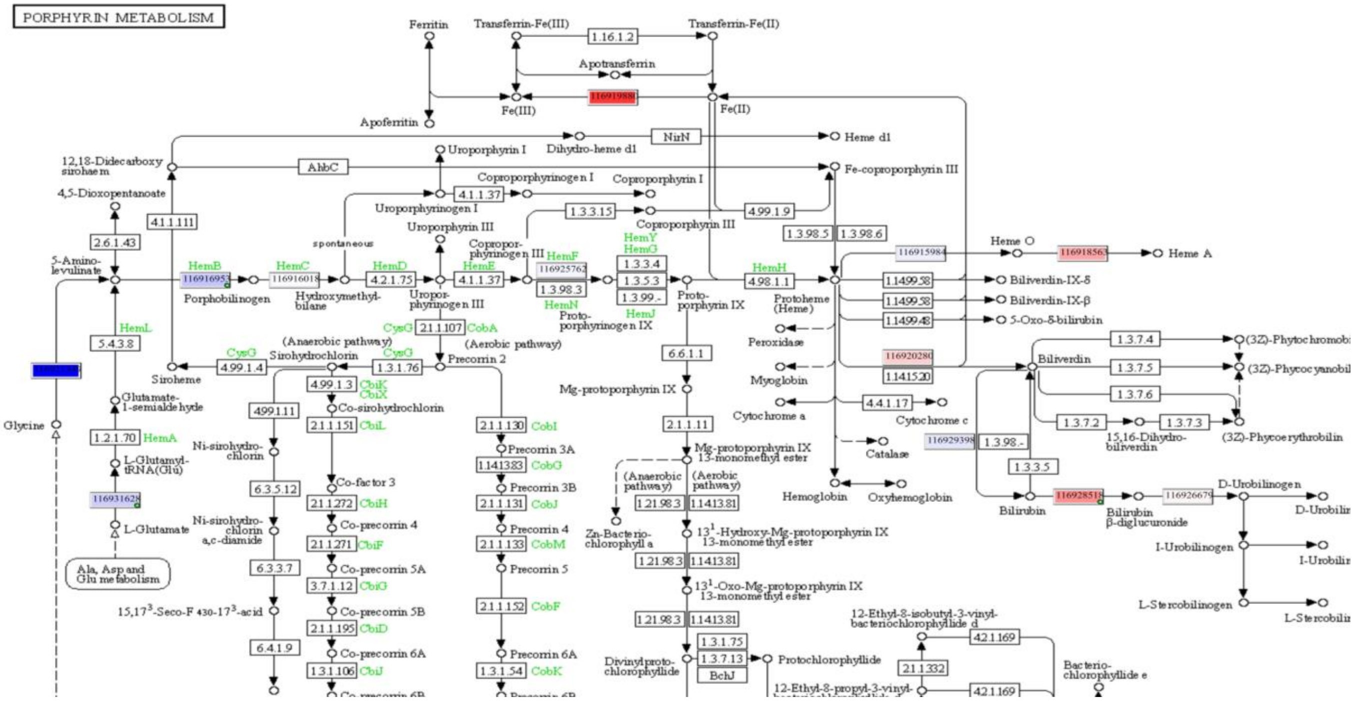
PORPHYRIN METABOLISM
Ferritin
Transferrin-Fe(III)
Transferrin-Fe(II)
1.16.1.2
Apotransferrin
Fe(III)
Fe(II)
Apoferritin
NirN
Dihydro-heme d1
Heme d1
Uroporphyrin I
12,18-Didecarboxy siroheam
AhbC
Fe-coproporphyrin III
4,5-Dioxopentanoate
Coproporphyrinogen I
Coproporphyrin I
Uroporphyrinogen I
Coproporphyrin III
Uroporphyrin III
5-Amino-levulinate
Porphobilinogen
Hydroxymethyl-bilane
Uropor-phyrinogen III
Proto-porphyrinogen IX
Proto-porphyrin IX
Protoheme (Heme)
Heme O
Heme A
Biliverdin
Bilirubin
Bilirubin β-diglucuronide
D-Urobilinogen
I-Urobilinogen
L-Stercobilinogen
Siroheme
Sirohydrochlorin
Precorrin 2
Glycine
L-Glutamate
Ala, Asp and Glu metabolism
Mg-protoporphyrin IX
Divinylproto-chlorophyllide
Protochlorophyllide
BchJ
Bacterio-chlorophyllide e
Precorrin 6A
Co-precorrin 6A
CbiJ
CobK

8 days

J

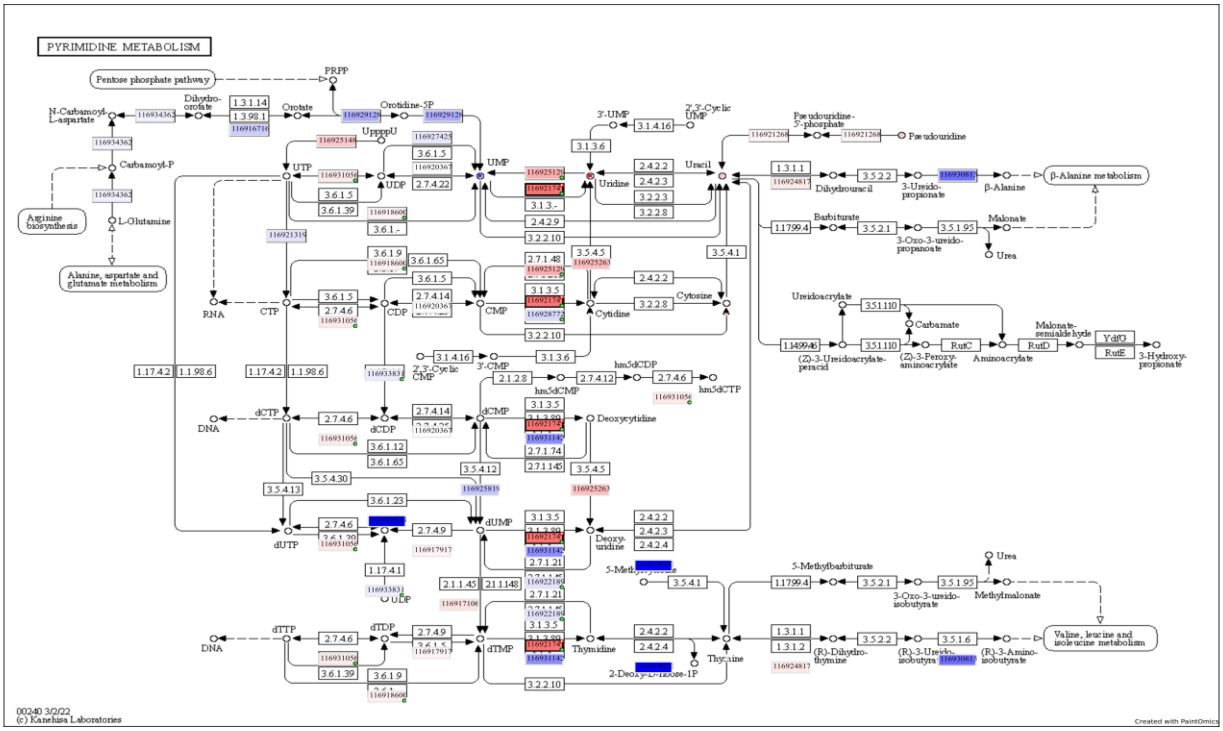

4 days

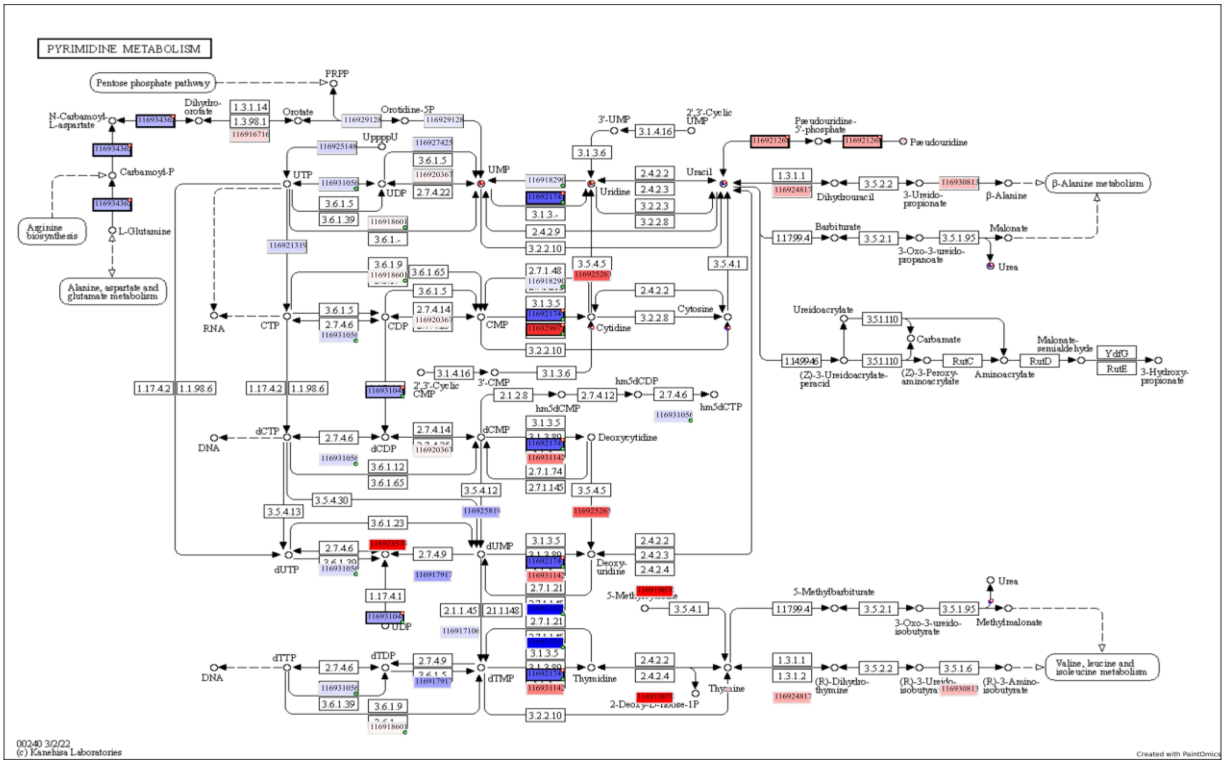

8 days

K

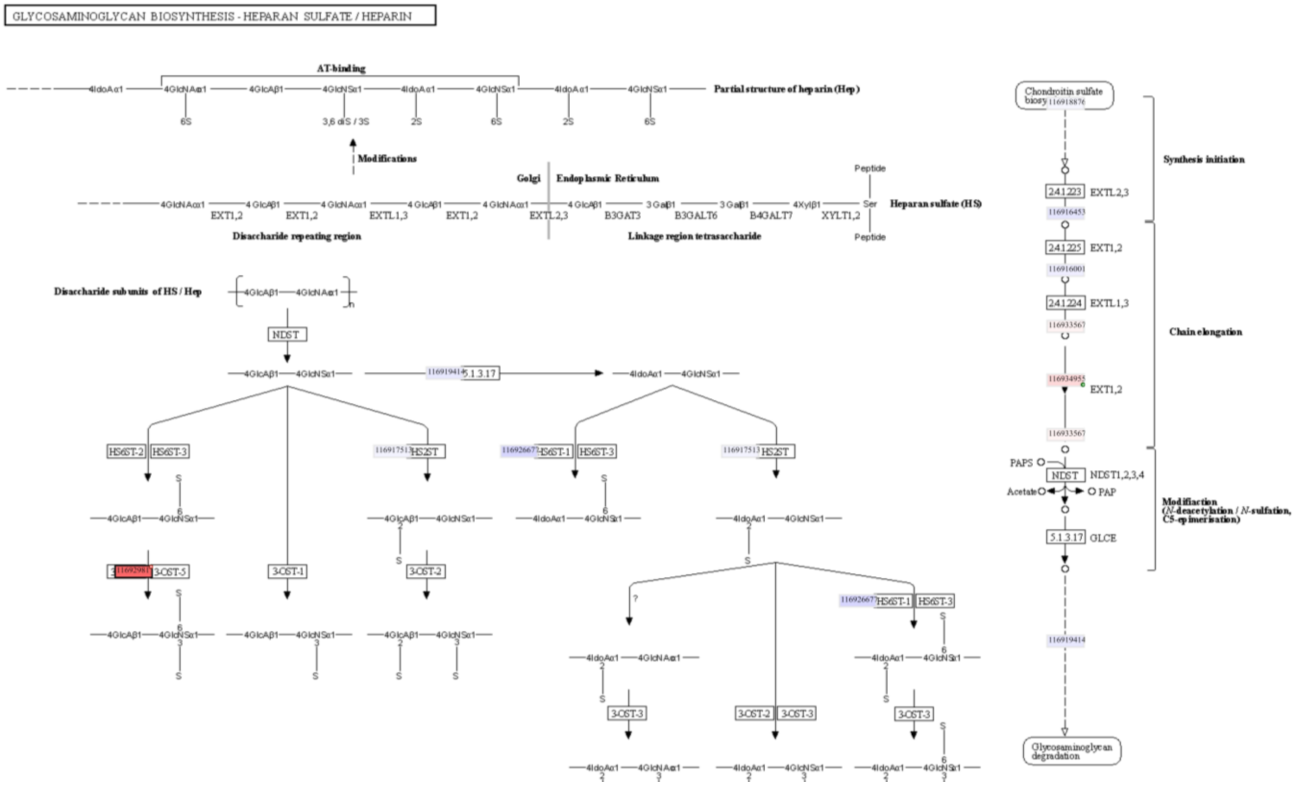

4days

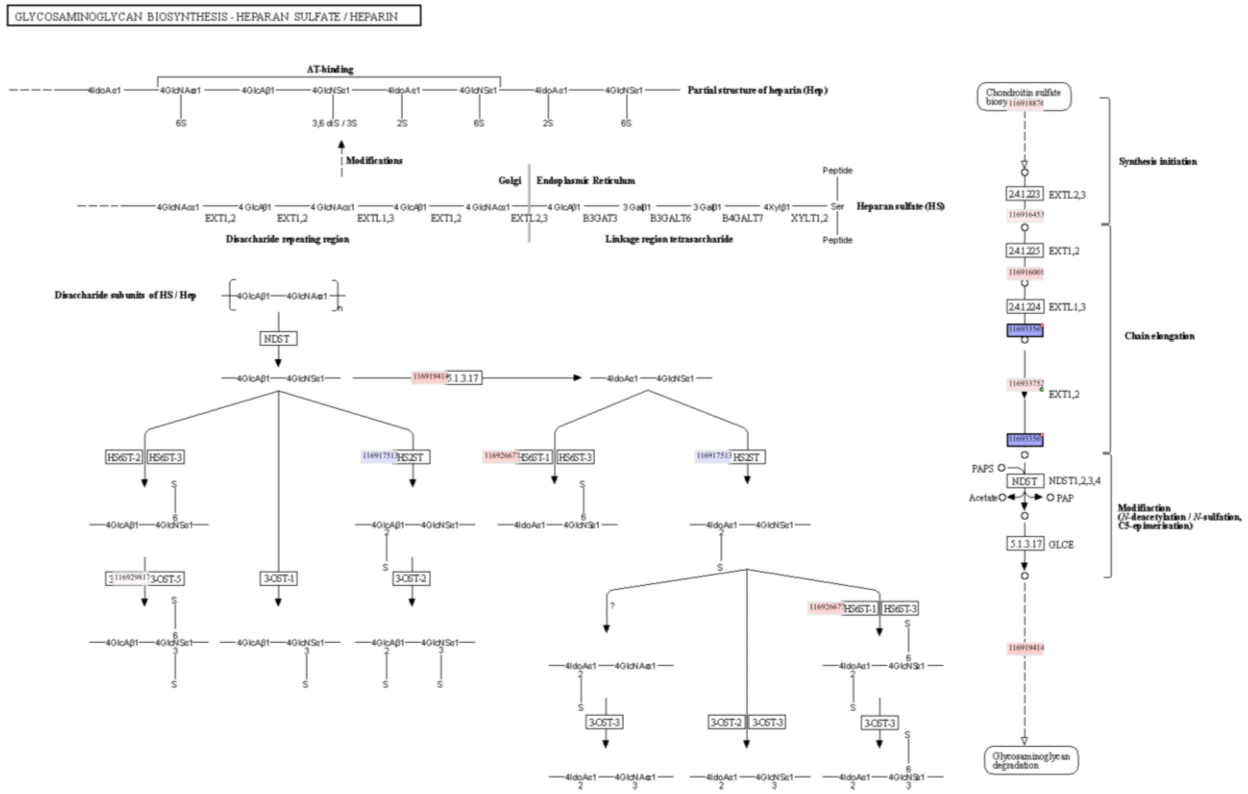

8 days

L

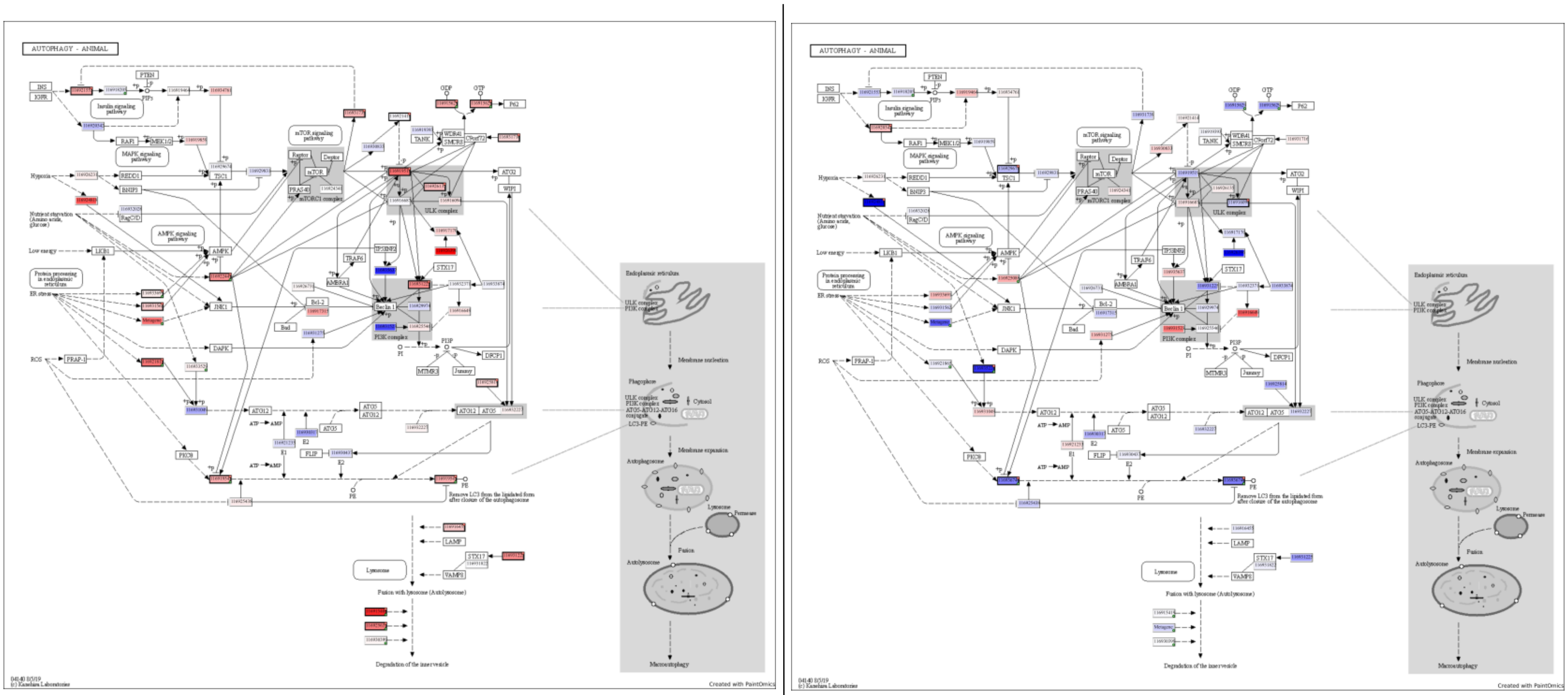
AUTOPHAGY - ANIMAL
AUTOPHAGY - ANIMAL

M

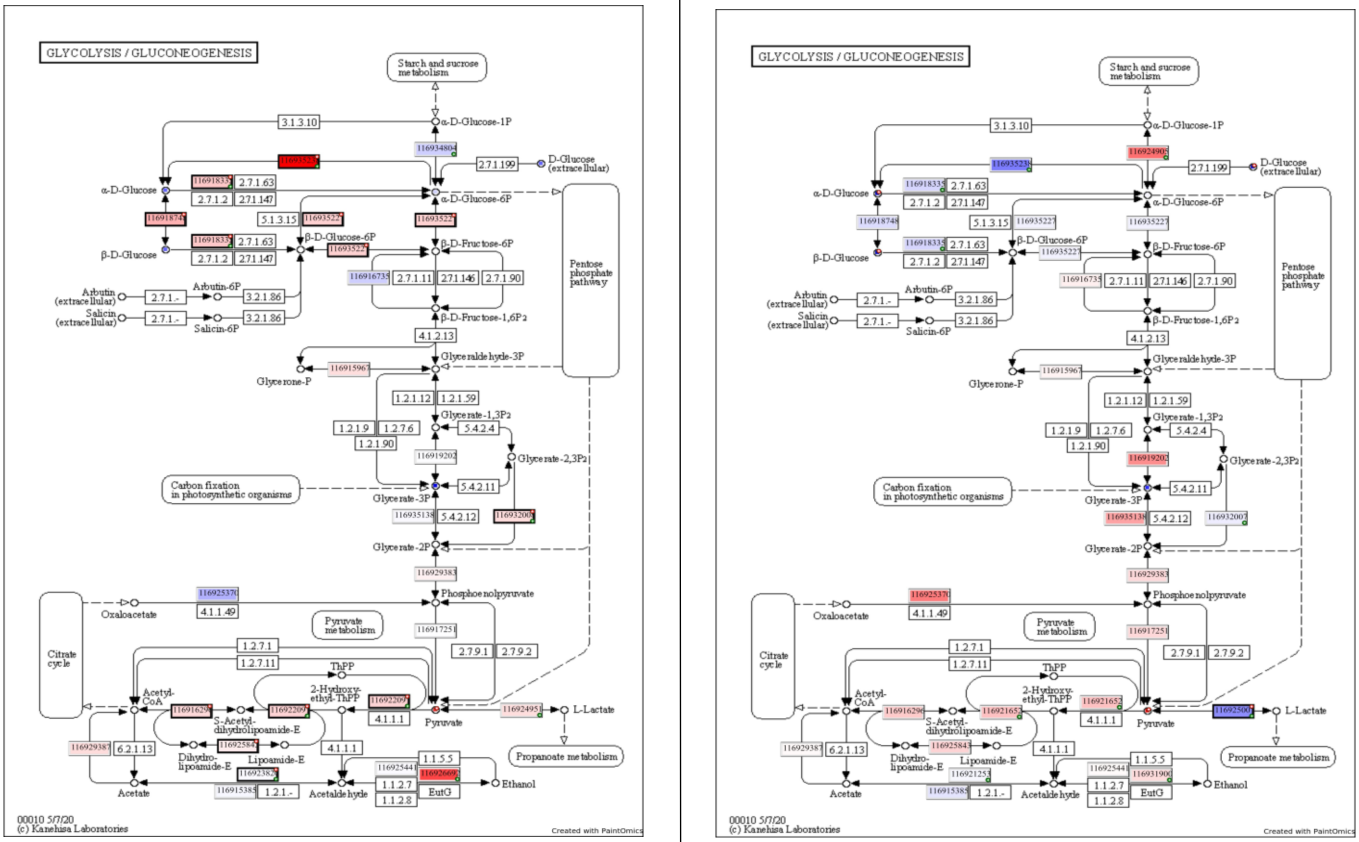
GLYCOLYSIS / GLUCONEOGENESIS
Starch and sucrose metabolism
α-D-Glucose-1P
D-Glucose (extracellular)
α-D-Glucose
α-D-Glucose-6P
β-D-Glucose
β-D-Glucose-6P
β-D-Fructose-6P
Pentose phosphate pathway
Arbutin (extracellular)
Salicin (extracellular)
Arbutin-6P
Salicin-6P
β-D-Fructose-1,6P2
Glyceraldehyde-3P
Glycerone-P
Glycerate-1,3P2
Glycerate-2,3P2
Carbon fixation in photosynthetic organisms
Glycerate-3P
Glycerate-2P
Phosphoenolpyruvate
Oxaloacetate
Pyruvate metabolism
Citrate cycle
Acetyl-CoA
S-Acetyl-dihydrolipoamide-E
2-Hydroxy-ethyl-ThPP
ThPP
Pyruvate
L-Lactate
Dihydro-lipoamide-E
Lipoamide-E
Propanoate metabolism
Acetate
Acetaldehyde
Ethanol
EutG
00010 5/7/20
(c) Kanehisa Laboratories
Created with PaintOmics
GLYCOLYSIS / GLUCONEOGENESIS

N

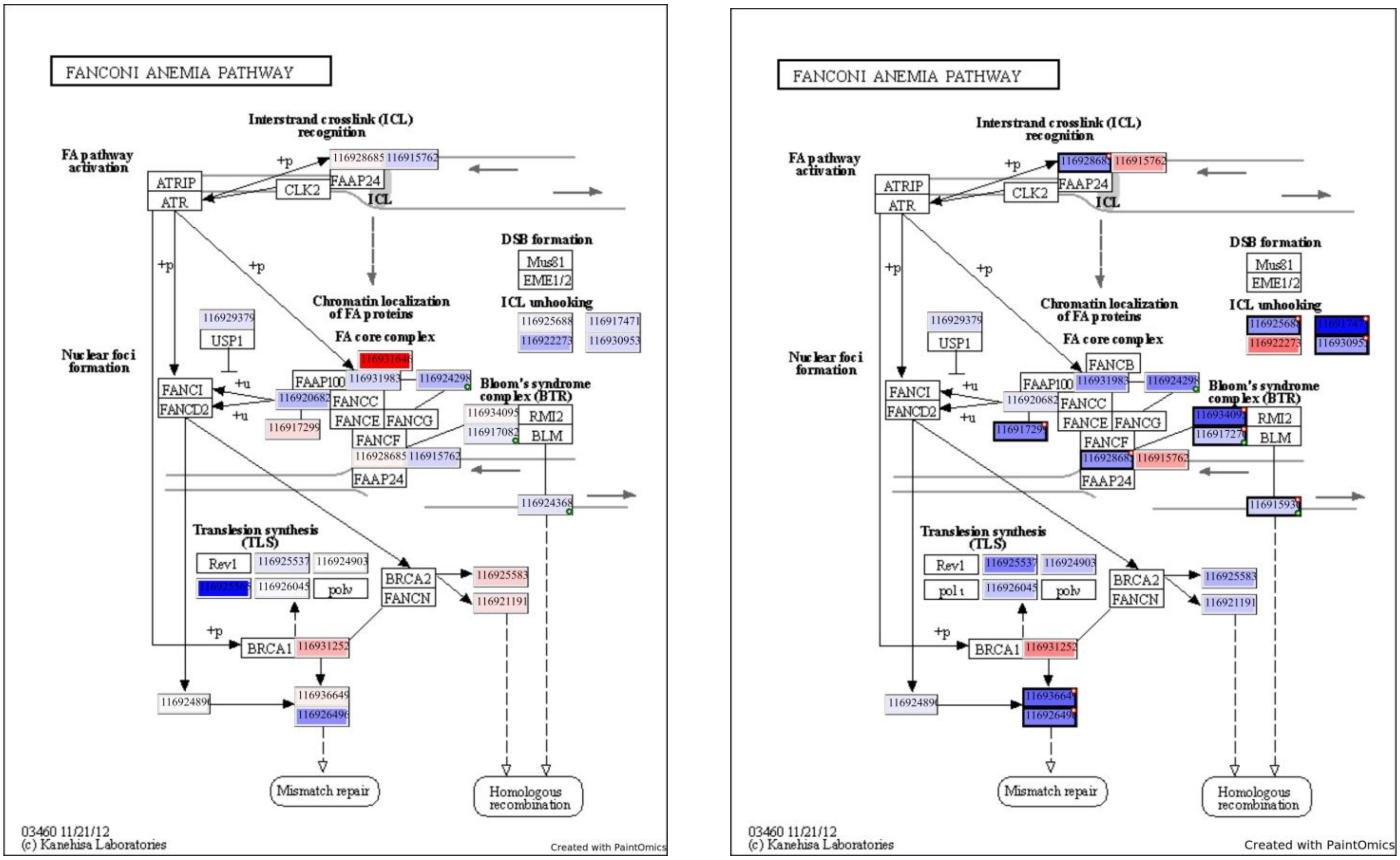
FANCONI ANEMIA PATHWAY
Interstrand crosslink (ICL) recognition
FA pathway activation
ATRIP
ATR
CLK2
FAAP24
ICL
DSB formation
Mus81
EME1/2
Chromatin localization of FA proteins
ICL unhooking
FA core complex
Nuclear foci formation
USP1
FANCI
FANCD2
FAAP100
FANCC
FANCE
FANCG
FANCF
FANCB
Bloom's syndrome complex (BTR)
RMI2
BLM
Translesion synthesis (TLS)
Rev1
polι
polν
BRCA2
FANCN
BRCA1
Mismatch repair
Homologous recombination
03460 11/21/12
(c) Kanehisa Laboratories
Created with PaintOmics

O

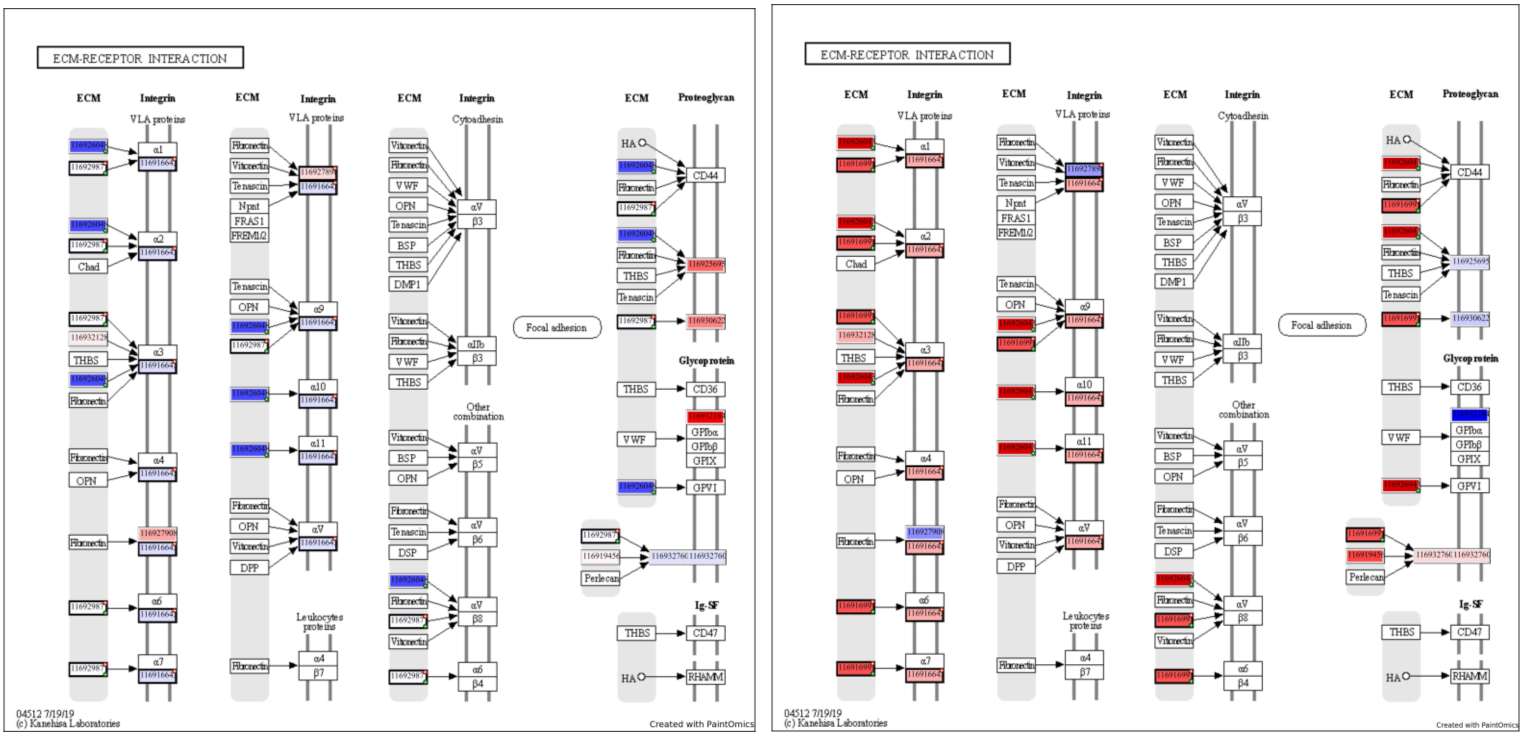
ECM-RECEPTOR INTERACTION
ECM
Integrin
VLA proteins
Cytoadhesin
Proteoglycan
Chad
THBS
OPN
Tenascin
Npnt
FRAS1
FREM1/2
Vitronectin
VWF
BSP
DMP1
DSP
DPP
HA
CD44
Perlecan
Focal adhesion
Glycoprotein
CD36
GPIbα
GPIbβ
GPIX
GPVI
Other combination
Leukocytes proteins
Ig-SF
CD47
RHAMM
04512 7/19/19
(c) Kanehisa Laboratories
Created with PaintOmics

P

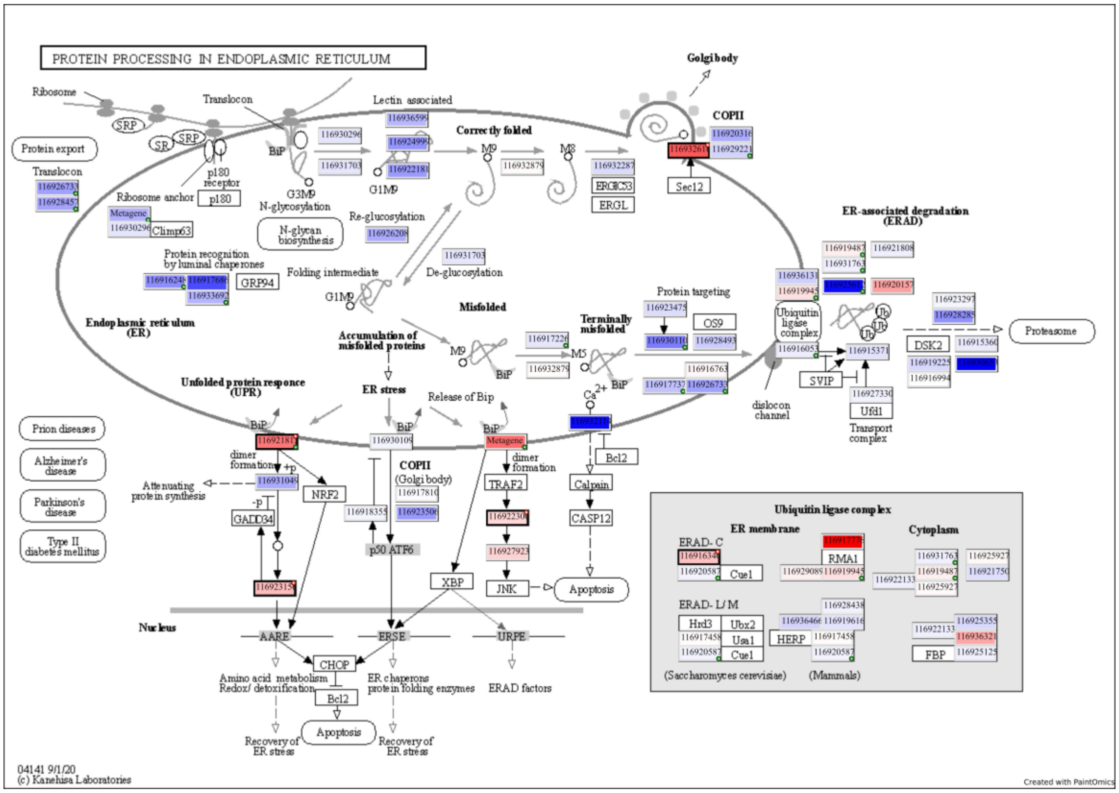
PROTEIN PROCESSING IN ENDOPLASMIC RETICULUM
Golgi body
Ribosome
Translocon
Lectin associated
Correctly folded
COPII
SRP
SR
Protein export
p180 receptor
p180
Ribosome anchor
Climp63
G3M9
N-glycosylation
G1M9
M9
M8
ERGIC53
ERGL
Sec12
Re-glucosylation
N-glycan biosynthesis
Protein recognition by luminal chaperones
GRP94
Folding intermediate
De-glucosylation
ER-associated degradation (ERAD)
Endoplasmic reticulum (ER)
Misfolded
Protein targeting
OS9
Terminally misfolded
Ubiquitin ligase complex
Proteasome
Accumulation of misfolded proteins
DSK2
SVIP
Ufd1
Transport complex
dislocon channel
Unfolded protein responce (UPR)
ER stress
Release of Bip
BiP
Metagene
Prion diseases
Alzheimer's disease
Parkinson's disease
Type II diabetes mellitus
dimer formation
Attenuating protein synthesis
NRF2
GADD34
COPII (Golgi body)
p50 ATF6
TRAF2
Calpain
CASP12
Bcl2
JNK
Apoptosis
XBP
Nucleus
AARE
ERSE
UPRE
CHOP
Amino acid metabolism Redox/ detoxification
ER chaperons protein folding enzymes
ERAD factors
Recovery of ER stress
Ubiquitin ligase complex
ER membrane
Cytoplasm
ERAD- C
RMA1
Cue1
ERAD- L/M
Hrd3
Ubx2
Usa1
HERP
FBP
(Saccharomyces cerevisiae)
(Mammals)
04141 9/1/20
(c) Kanehisa Laboratories
Created with PaintOmics

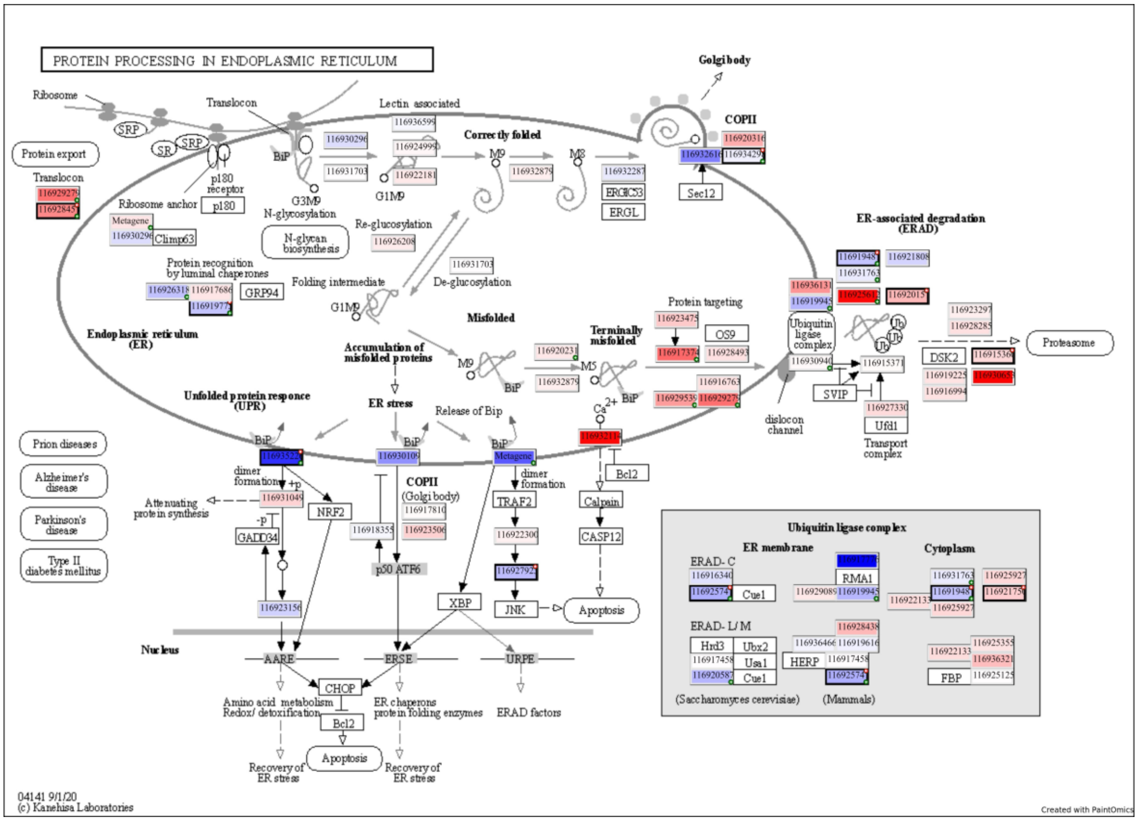
PROTEIN PROCESSING IN ENDOPLASMIC RETICULUM
Golgi body
Ribosome
Translocon
Lectin associated
Correctly folded
COPII
SRP
SR
Protein export
p180 receptor
p180
Ribosome anchor
Climp63
G3M9
N-glycosylation
G1M9
M9
M8
ERGIC53
ERGL
Sec12
Re-glucosylation
N-glycan biosynthesis
Protein recognition by luminal chaperones
GRP94
Folding intermediate
De-glucosylation
ER-associated degradation (ERAD)
Endoplasmic reticulum (ER)
Misfolded
Protein targeting
OS9
Terminally misfolded
Ubiquitin ligase complex
Proteasome
Accumulation of misfolded proteins
DSK2
SVIP
Ufd1
Transport complex
dislocon channel
Unfolded protein responce (UPR)
ER stress
Release of Bip
BiP
Metagene
Prion diseases
Alzheimer's disease
Parkinson's disease
Type II diabetes mellitus
dimer formation
Attenuating protein synthesis
NRF2
GADD34
COPII (Golgi body)
p50 ATF6
TRAF2
Calpain
CASP12
Bcl2
JNK
Apoptosis
XBP
Nucleus
AARE
ERSE
UPRE
CHOP
Amino acid metabolism Redox/ detoxification
ER chaperons protein folding enzymes
ERAD factors
Recovery of ER stress
Ubiquitin ligase complex
ER membrane
Cytoplasm
ERAD- C
RMA1
Cue1
ERAD- L/M
Hrd3
Ubx2
Usa1
HERP
FBP
(Saccharomyces cerevisiae)
(Mammals)
04141 9/1/20
(c) Kanehisa Laboratories
Created with PaintOmics

Q

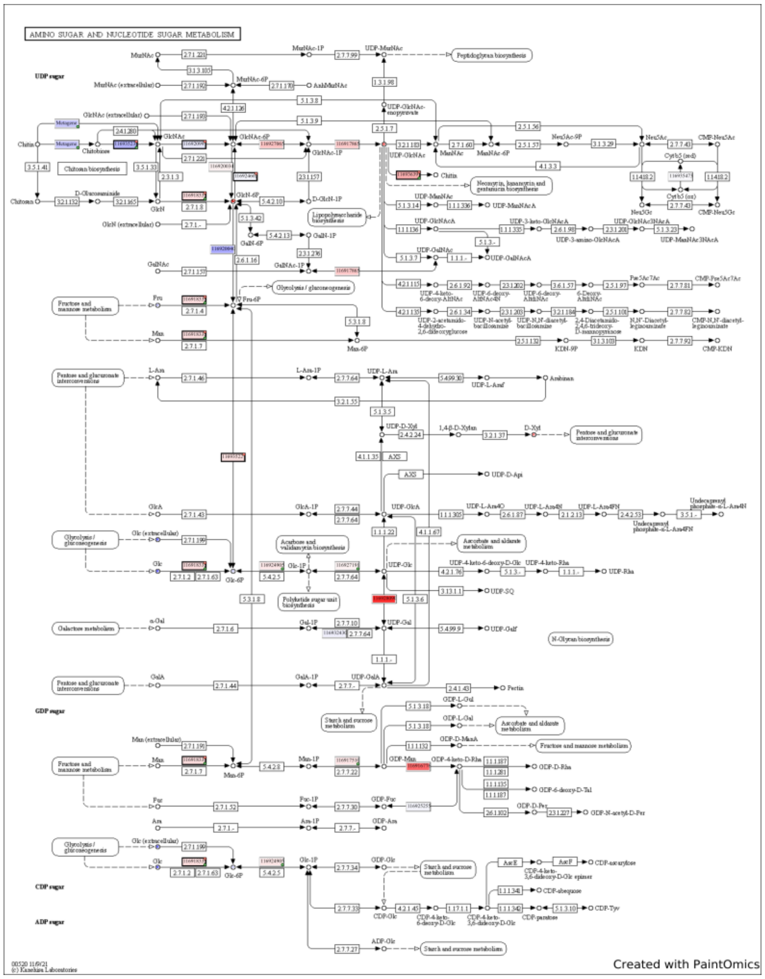

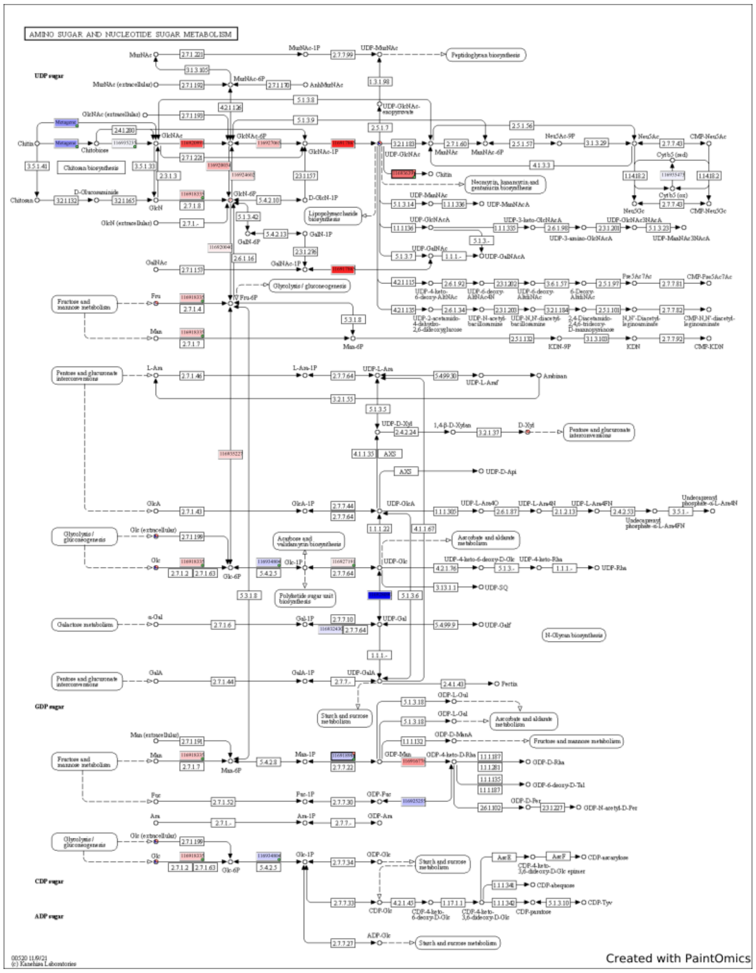

Table S4: *DiCE genes and their corresponding NCBI gene identification.*

DiCE genes	NCBI gene id
VTG-SOD	LOC116916592 , LOC116917901
ESR16	LOC116924588
Dhb1	LOC116926535 , LOC116926537
VTG2	LOC116927135
VTG2 (isoforms)	LOC116922763, LOC116928002, LOC116934410 and LOC116934974

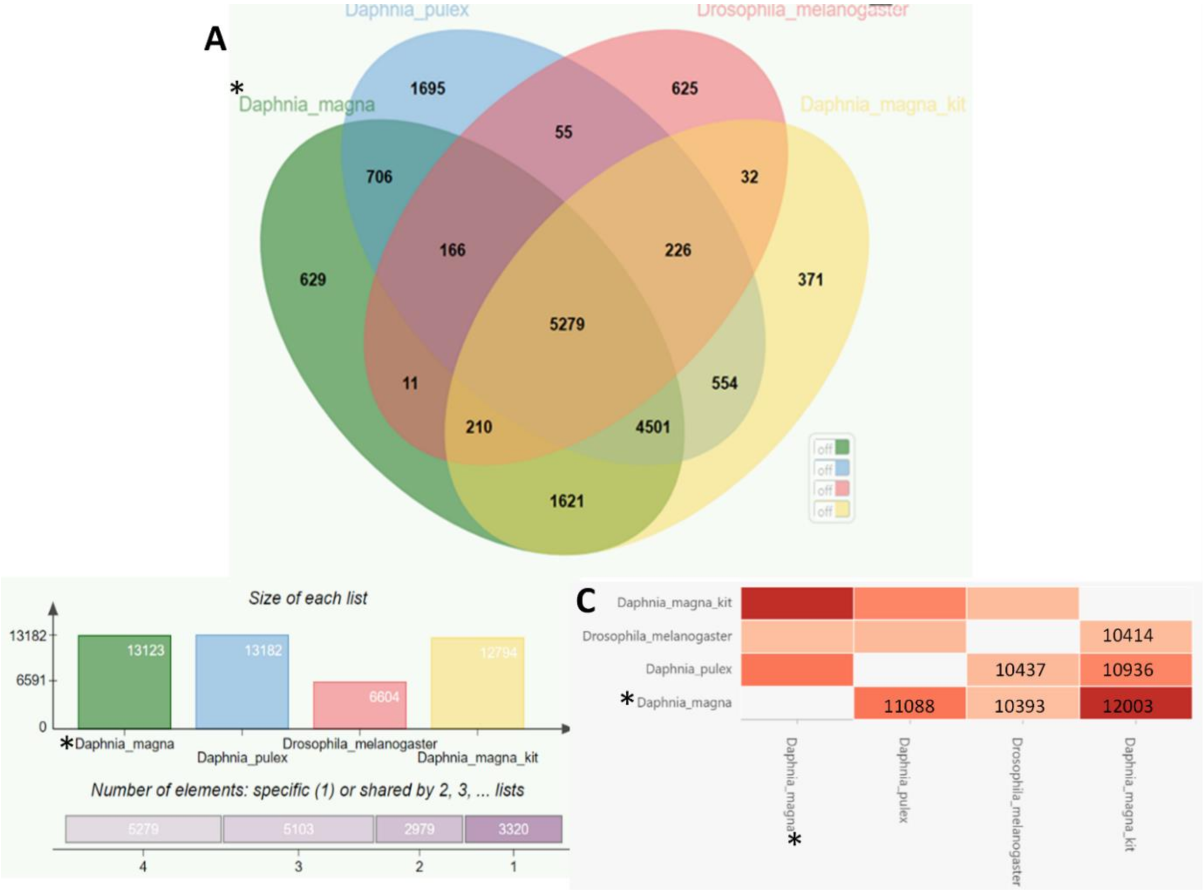

Fig. S11: *Comparison of orthologous genes between different clones of Daphnia magna, Daphnia pulex and Drosophila melanogaster. A: Venn diagram showing the numbers of shared orthologous groups between D. pulex, D. magna xinb3, D. magna KIT and D. melanogaster. B: The bar graph above shows the numbers of protein clusters found in each species, while the bar plot below displays the number of orthologous clusters shared by 1, 2, 3 and 4 species. C: Pairwise heatmap with number of overlapping clusters between different pairs of species. The overlapping cluster numbers were indicated in the cells and the colour intensity followed the shared number of orthologous groups: the darker the colour, the more orthologs shared between species.*

**Daphnia_magna labelled with asterisk (*) sign refers to Daphnia magna xinb3, a different strain from our study species D.magna KIT.*
